# The Current State of Global Awareness and Knowledge on Oral Cancer: A Narrative Review

**DOI:** 10.1002/hsr2.71834

**Published:** 2026-02-18

**Authors:** Kehinde Kazeem Kanmodi, Yovanthi Anurangi Jayasinghe, Ruwan Duminda Jayasinghe, Emeka Benjamin Okeke, Misheck Julian Nkhata, Lawrence Achilles Nnyanzi

**Affiliations:** ^1^ School of Health and Life Sciences Teesside University Middlesbrough UK; ^2^ Centre for Dental and Craniofacial Research Cephas Health Research Initiative Inc. Ibadan Nigeria; ^3^ Department of Public Health Thomas Adewumi University Oko Nigeria; ^4^ Centre for Evidence Synthesis and Implementation Research, Cephas Health Research Initiative Inc. Ibadan Nigeria; ^5^ Department of Oral Medicine and Periodontology University of Peradeniya Peradeniya Sri Lanka; ^6^ Centre for Digital Health Research, Innovation and Practice, Cephas Health Research Initiative Inc. Ibadan Nigeria

**Keywords:** awareness, diagnosis, future directions, information sources, knowledge, oral cancer, prevention, review, risk factors, treatment

## Abstract

**Background and Aims:**

Limited public knowledge of oral cancer often results in late‐stage diagnosis, contributing to its status as a global health burden. This narrative review aims to systematically pool gather evidence on current information sources and current state of awareness and knowledge on definition, risk factors, clinical features, prevention, diagnosis and treatment of oral cancer, to identify knowledge gaps and public health implications.

**Methods:**

This narrative review relied on secondary data obtained from relevant literature obtained from PubMed, Google Scholar, and websites of reputable health organizations. Only those literatures published in English were utilized, and most of them were published within the past 5 years to ensure that the evidence synthesised in this review are based on contemporary evidence.

**Results:**

Most of the obtained research focused on Asian populations, community members, students, and patients, with limited attention to healthcare professionals and high‐risk groups like farmers and sex workers. The preferred information sources on oral cancer included digital media (TV, internet, radio) and non‐digital channels (healthcare workers, newspapers), though the latter remained more commonly used despite the growing potential of digital tools. Awareness of oral cancer's definition varied widely (15%–99%) with the highest levels observed among medical/dental students. Knowledge on established oral cancer risk factors (e.g. tobacco, alcohol, human papillomavirus) was inconsistent, with misinformation commonly observed among lay populations. Non‐healing ulcers and bleeding were reported as poorly recognized clinical features, even among professionals, while pain was notably underreported. Knowledge on prevention (e.g. smoking cessation and screening) was generally low, and awareness of diagnostic tools (e.g. biopsies) were limited to health trainees in Asia. Awareness of treatment options varied, with limited understanding of the roles of specialist healthcare providers.

**Conclusion:**

The findings of this review highlight the importance of more inclusive research to guide targeted interventions on oral cancer.

## 1 Introduction

Oral cancer refers to a malignant neoplasm which occurs in the lip, oral cavity—tongue, buccal mucosa, floor of the mouth, soft and hard palate and gingiva— and the oropharynx, where majority of the oral cancer cases presenting as squamous cell carcinomas [1, 2]. Oral cancer poses a significant global health burden. According to the Global Cancer Observatory, GLOBOCAN, oral cancer is ranked as the 16th most common cancer worldwide, with an estimated 389,846 new cases and 188,432 mortalities reported in the year 2022 [3]. Among these incidences a substantial proportion of oral cancer cases are reported from low‐ and middle‐income countries, where the disease burden remains disproportionately high [4, 5]. Despite advancements in diagnosis and treatment, oral cancer is frequently detected at advanced stages often due to the absence of early symptoms and delays in diagnosis [6]. According to several systematic and scoping reviews, a key contributor to this delay is the limited public awareness and understanding of oral cancer, its risk factors and early warning signs [7, 8, 9]. Thereby, this narrative review aims to explore the current state of global awareness and knowledge on oral cancer, identify key gaps in public understanding and highlight the need for enhanced health education and awareness strategies.

Over the years, several empirical studies had explored awareness and knowledge rate on oral cancer. To date, no study has provided a global synthesis of public awareness and knowledge on oral cancer [10]. Regardless of the paucity of such a robust study with a global scope, it was nonetheless identified that the global research landscape on awareness and knowledge on oral cancer has been dynamic over the past six decades [10, 11, 12]. So far, awareness and knowledge of different aspects of oral cancer—including oral cancer definition/meaning, its risk factors, clinical features, preventability, preventative strategies (or measures), diagnosis, treatability, and treatment—have been explored extensively through several surveys. This shows that research on oral cancer literacy is an area of keen interest across researchers across the world.

As evidenced on the PubMed database—the largest electronic research database for medical and health science literature, the oldest literature reporting an investigation on the awareness or knowledge on oral cancer was a peer‐reviewed journal article which was published in the year 1973, by Pullon and Miller, and it was published in the journal entitled “Journal of American Dental Association” [13]. The study was on a 1971 survey (i.e. data was collected in the year 1971) of dentists in the State of Pennsylvania, United States, to evaluate their collective knowledge on the rates of survival of early‐ and advanced‐stage oral cancer. The study reported that the majority of Pennsylvania dentists had pessimistic and erroneous predictions on these survival rates [13]. To the best of the researcher's knowledge, this study is perhaps the oldest peer‐reviewed original research article publication on oral cancer knowledge/awareness all over the world.

After this, the second oldest study was another American study which was published as a peer‐reviewed original research journal article in the year 1988 [14]. Like the oldest known study [13], this study was also conducted among dentists. In the study, it could not be validated that dentists’ knowledge is associated with their behaviour on oral cancer case‐finding; however, it was substantiated in the study that knowledge of confirmed oral precancer or oral cancer diagnosis was associated with dentists’ oral precancer or oral cancer case‐finding behaviour [14].

**Figure 1:**
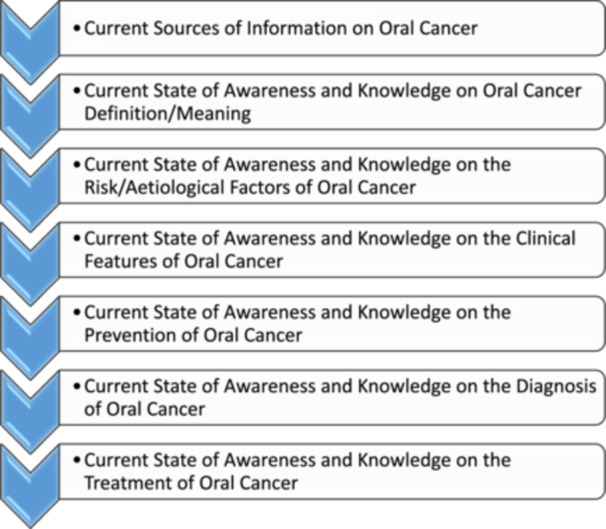
Schematic description of the flow of the subsections discussing the state of global awareness and knowledge on oral cancer.

With emphasis, there is a low prospect in providing a robust overview on the trajectory of global knowledge and awareness rates on oral cancer due to the current lack of a robust multi‐country/multi‐continental cross‐sectional/longitudinal survey on the level of global knowledge and awareness on oral cancer, and this justifies why this section of this literature review focused on the current state of global awareness and knowledge on oral cancer. Hence, the evidence presented in this section were based on surveys published on oral cancer awareness and knowledge published from 2020 to 2024. To ensure adequacy, two databases (PubMed and Google Scholar) were searched. The findings are presented under thematic subsections. The figure below gives a schematic description of the flow of these subheadings (Figure 1).

## 2 Literature Source

This narrative review adopted the reporting style used in similar reviews [15, 16, 17, 18], and it relied on secondary data obtained from relevant literature obtained from PubMed, Google Scholar, and websites of reputable health organizations (including National Health Service, Centres for Disease Control and Prevention, World Health Organization, and Africa Centre for Disease Control and Prevention). Only those literatures published in English and whose content are relevant to the objectives of this narrative review were utilized, and they were published within the past 5 years to ensure that the evidence synthesised in this review are based on contemporary evidence. The search terms used to identify the literature utilised in this narrative review included ‘oral cancer’, ‘know*’, and ‘aware*’, and those contemporary literature published within the past 5 years (2020 to 2024) on awareness and knowledge of oral cancer were the primary focus, although some few older literature were used to critically discuss the evidence synthesised from those contemporary literature.

### 2.1 Current Sources of Information on Oral Cancer

Before discussing the current state of awareness and knowledge on the definition/meaning, risk/aetiological factors, clinical features, prevention, and treatment of oral cancer, it is crucial to first discuss the sources utilised across the world to obtain information on oral cancer in the current (or contemporary) literature. A comprehensive review of this information will provide deep insights on the nature and public accessibility to these sources; also, in this section, recent literature was defined as studies published between 2020 and 2024.

Between 2020 and 2024, we found sixteen peer‐reviewed articles that have surveyed different populations of the world on their sources of information on oral cancer [19, 20, 21, 22, 23, 24, 25, 26, 27, 28, 29, 30, 31, 32, 33, 34] (Table S1). Notably, these articles reported findings on populations in all continents of the world except Africa—that is, no relevant article was found to have been published on African populations within the past half‐decade. Evidently, this identified evidence gap is an issue of public health concern, as it is of paramount importance that the current sources of information on oral cancer among African populations are known as well, as such information will provide deep insights on the level of credibility and the potential risks of misinformation on oral cancer among the African populace [35, 36, 37, 38].

Based on evidence reported in these contemporary articles, it was observed that the world populations utilise diverse sources to obtain information on oral cancer. These information sources could be grouped into digital and non‐digital sources [39]. The digital sources include internet [19, 21, 24, 26, 31, 32], radio [21, 23, 25, 31, 32], television [19, 21, 23, 25, 27, 31], and social media [27, 34]. Of all these digital sources, the internet (range of usage rate: 14.0% to 53.8%), radio (range of usage rate: 3.8% to 33.1%), and television (range of usage rate: 14.2% to 63.5%) were the three most common sources used across diverse populations of the world.

Importantly, emerging digital sources of information such as mobile health applications [39, 40] and artificial intelligence [40, 41] were not reported among the participants of the reviewed articles. This shows that despite the rapid growth of information technology, the uptake of emerging technologies by the general public as a main or supporting source of information on oral cancer remains slow. These two emerging digital sources of information, namely mobile health applications and artificial intelligence, are considered more effective, reliable, and user‐friendly compared to other sources [39, 40, 41]. This therefore demonstrates the need to promote the use of these emerging sources globally.

Concerning the non‐digital sources of information on oral cancer, this could be sub‐categorised into human sources and non‐human sources. The human sources sub‐category can further be grouped into healthcare professionals and non‐healthcare professionals. Notably, medical doctors [19, 21, 22, 25, 28, 30, 31, 32], dentists [19, 21, 24, 25, 28, 30, 32, 33], nurses [22], midwives [25], and pharmacists [32], are the healthcare professionals that were predominantly consulted by the general populations for information concerning oral cancer.

Among these healthcare professionals, dentists (range of usage rate: 5.8% to 51.0%) and medical doctors (range of usage rate: 4.8% to 31.0%) were the top two most utilised sources. The relatively higher level of clinical and/or public health competence of dentists and medical doctors on oral cancer might have been a factor encouraging the utilisation of these two healthcare professionals as sources of information on oral cancer among diverse populations. Unfortunately, not all countries and regions of the world had adequate supply of dentists and medical doctors [36]; for instance, as low as 1% of the 1.6 million dentists in the world are in Africa [42]. By implication, this means that inequalities exist in the global access to these two healthcare professionals for information on oral cancer [36]. To reduce these existing inequalities, it becomes imperative that other healthcare professionals in low‐resource settings, such as in many African countries, are trained to educate the public about oral cancer.

On the other hand, the non‐healthcare professionals that were utilised sources of information on oral cancer include friends [19, 21, 22, 25, 27, 31, 33], colleagues [21], neighbours [27], and relatives (or family members) [21, 22, 24, 25, 28, 33]. Notably, of these four sources, the top two most utilised sources were friends (range of usage rate: 1.6% to 47.0%) and relatives (range of usage rate: 1.6% to 47.0%). By implication, these identified findings suggest that other than sourcing information from healthcare professionals, people also consult their loved ones for information on oral cancer.

The non‐human non‐digital category of sources of information on oral cancer include newspapers [19, 21, 23, 25, 26, 31, 32], magazines [21, 23, 26, 32], cigarette packages [21], academic institutions of learning [20, 22, 29], religious institutions [22], social or academic or public events (e.g. health campaigns, scientific meetings) [20, 22, 24, 29], posters [25, 26], banners [25, 26], journals [29], and books/textbooks [23, 29]. However, of all these non‐human non‐digital sources, the top two most utilised sources were newspapers (range of usage: 22.3% to 44.2%) and magazines (range of usage rate: 5.3% to 44.2%). Based on common knowledge, these two information sources—newspapers and magazines—have larger reach across populations compared to the other non‐human non‐digital source types; this therefore explains the popularity of these two sources as a source of information on oral cancer.

Although non‐digital channels remain more frequently used, the expanding reach of digital platforms creates an opportunity to strengthen public education through credible online and mobile health resources [39, 43].

### 2.2 Current State of Awareness and Knowledge on Oral Cancer Definition/Meaning

The understanding of the current state of global awareness and knowledge on the definition/meaning of oral cancer is very crucial, as this provides insight on what the public knows about the meaning of the disease, as this will help in identifying the problem of misinformation across the world populations. The findings reported in this section were based on twenty‐six contemporary peer‐reviewed articles which investigated public awareness and knowledge of the term oral cancer (including its sub‐types) among diverse population groups (Table S2) [19, 22, 24, 27, 28, 30, 33, 44, 45, 46, 47, 48, 49, 50, 51, 52, 53, 54, 55, 56, 57, 58, 59, 60, 61, 62].

Notably, majority of these articles only reported findings on the populations in Asian and European countries, while studies on populations in countries in other continents of the world were either negligible or absent. This paucity of information on the awareness and knowledge on the definition/meaning of oral cancer suggests the need for current research on this topic area, especially on populations in Africa, North America, South America, and Australia and the Oceania which have a huge dearth of contemporary evidence on the topic area.

Furthermore, these reviewed articles investigated community dwellers, students, and patients while a few were conducted among university visitors, dentists, and military personnel. The relatively higher frequency of studies investigating community dwellers, students, and patients, compared to those investigating other population groups (including healthcare professionals, university visitors, and military personnel) is not so surprising, given the fact that community dwellers, students, and patients are relatively more accessible to researchers compared to the other population groups. Notably, populations of healthcare professionals (such as medical doctors, nurses, midwives, dental hygienists, and pharmacists) and those peculiar population groups at risk of oral cancer (such as commercial sexual workers, industrial workers, and farmers) were not among the primary target populations of these contemporary articles [63, 64]. These findings highlight the underrepresentation of healthcare professionals and high‐risk groups in current studies. Future investigations should generate population‐specific evidence to guide tailored interventions [65, 66].

Based on the findings reported among community dwellers and patients while a few were conducted among university visitors, dentists, and military personnel, it can be estimated that the current global prevalence of awareness and knowledge of the term “oral cancer” (or its subtype) ranged from 15.35% to 99.0%. However, these prevalence rates vary across different population groups. Among community dwellers, the prevalence ranged from 47.05% to 87.3%; among dentists, it ranged from 88.07% to 90.2%; among patients, it ranged from 20% to 80%; among dental/medical students, it ranged from 86.3% to 99.5%; among non‐dental/non‐medical students, it ranged from 41% to 66%; among university visitors, it was 68.4%; and among military personnel, it was 15.35%.

The observed disparities in the prevalence of awareness observed in these diverse population groups demonstrates varying levels of awareness on oral cancer based on population peculiarities. Dental students, medical students, and dentists, all of whom are in the fields of healthcare, were found to be population groups with the highest and narrowest ranges of awareness rates on the term oral cancer. On the other hand, for other population groups, such as community dwellers, patients, non‐dental and non‐medical students, university visitors, and military personnels, wider ranges and/or lower prevalence of awareness on the term oral cancer was observed. These disparities are not too surprising because it is expected that clinicians and clinical students, compared to any other population groups, should be more knowledgeable about oral cancer, due to their exposure to clinical and academic training opportunities where they can acquire robust knowledge on oral cancer.

Furthermore, only one of these reviewed articles reported the knowledge of the term oral cancer (including its sub‐types) investigated if their participants had knowledge of the case definition of oral cancer [53]. In the study, by Jafer et al. [53], only 81.7% of their participants knew that oral cancer is a malignant disease while only 59% knew that oral cancer can metastasise to other body parts. This identified gap in the reviewed articles demonstrates the need for further empirical studies investigating public knowledge of the case definition of oral cancer. Conducting such further studies are of paramount importance because having awareness of oral cancer does not necessarily translate to having actual knowledge of it, and multiple literature has confirmed that several misconceptions concerning oral cancer exists among the lay populations [40].

Only seven of these articles identified the determinants of the awareness/knowledge of the term oral cancer (including its sub‐types) [24, 27, 33, 44, 45, 55, 57]. This finding indicates that most of the contemporary articles on the topic area lacked robust statistical analysis, as statistical analysis of determinants (such as determination of odds ratios) provides a more reliable information on those factors that plays influential role concerning a state of health [67].

The factors that were consistently found, across different populations, as significant determinants of knowledge of the term oral cancer (including its sub‐types) includes participants' source of information on oral cancer [57], course of study [44], ethnicity [24], occupation [24, 57], history of dental visit [55], alcohol use history [55], history of use of betel nut [27], location of residence [27, 57], average household monthly income [57], acquaintance with someone with cancer [57], mouth self‐examination practices [27], and school type [45]. On the other hand, marital status [57] was the only determinant that was consistently not significant.

However, factors such as age [44, 45, 55, 57], sex/gender [33, 44, 45, 55, 57], smoking history [55, 57], level of educational attainment [33, 45, 55, 57], were found to be disputable determinants of awareness and knowledge of the term oral cancer (including its sub‐types), as some studies [33, 45, 55, 57] reported that they were significant factors while some [44, 45, 57] reported that they were not significant. Since some of the determinants of awareness and knowledge of the term oral cancer (including its sub‐types) were consistently significant while some were disputable, it can be suggested that a meta‐analysis is further conducted on those determinants that were found to be disputable, as findings from such meta‐analysis will provide a more concrete analysis which will lend further insights on the applicability of such factors in the planning, development, and implementations of educational interventions on oral cancer targeting people associated with such factors [68].

### 2.3 Current State of Awareness and Knowledge on the Risk/Aetiological Factors of Oral Cancer

The understanding of the current state of global awareness and knowledge on the risk/aetiological factors of oral cancer is very crucial, as this provides insight on what the public knows about what causes the disease, as this will help in identifying the problem of misinformation across the world populations. For this sub‐section, sixty‐two contemporary peer‐reviewed articles which reported awareness and knowledge on the risk/aetiological factors of oral cancer among diverse population groups of the world were used to build the body of information presented in this section (Table S3) [19, 20, 21, 22, 23, 24, 27, 28, 29, 30, 31, 32, 33, 44, 46, 47, 48, 49, 50, 51, 52, 53, 54, 57, 58, 59, 61, 62, 69, 70, 71, 72, 73, 74, 75, 76, 77, 78, 79, 80, 81, 82, 83, 84, 85, 86, 87, 88, 89, 90, 91, 92, 93, 94, 95, 96, 97, 98, 99, 100, 101, 102].

Notably, these articles investigated populations from fourteen Asian countries, eight European countries, two African countries, two North American countries, one South American country and one country in Australia and Oceania. These findings show that evidence is abundant in Asia and Europe but sparse in other continents. Furthermore, most of these articles predominantly focused on community dwellers, patients, students, and dentists. Asides community dwellers, patients, students, and dentists, only very few of them focused on healthcare professionals and other groups. The healthcare professionals that were investigated in these few articles were medical doctors [82], nurses [82], healthcare assistants [82], and dental hygienists [100] while the other groups were farmers [78], military personnel [58], university visitors [33], and healthy relatives of patients [23].

The disproportionately high level of focus of these contemporary articles on community dwellers, patients, students, and dentists, compared to other population groups is, possibly, an indication of low research interest on the topic area outside the afore‐mentioned population groups. Unfortunately, farmers constitute a population at high risk of lip cancer (an oral cancer type) due to their outdoor farming activities which increases their risks to prolonged exposure to ultraviolet radiation from the sun [103]. Also, it is also important that the prevalence of awareness and knowledge of healthcare professionals on the risk or aetiological factors of oral cancer is known; this is because healthcare professionals have been found to be a major human source of information on oral cancer (see the preceding sub‐section on current sources of information on oral cancer), and more countries are now considering the need to engage non‐dental healthcare professionals to support the provision of oral health preventative (including health education services) in order to boost oral health (including oral cancer) literacy among their lay populations [104, 105, 106]. This evidence gap suggests upcoming studies should prioritise under‐researched groups, including farmers and non‐dental healthcare professionals.

Notably, several risk/aetiological factors of oral cancer were known to the participants in these reviewed contemporary articles. These factors include occupational factors (exposure to sunlight or ultraviolet radiation), dietary factors (consumption of hot and/or spicy foods or drinks, and poor nutrition and diets), pathogenic factors (human papillomavirus infections and other viral disease infections), behavioural and socio‐economic factors (excessive chewing of gums, use of cell phone, sexual activities, alcohol use, tobacco use, use of other recreational preparations [such as bidis, gutka, pan masala, etc.], and low socio‐economic factors), genetic or hereditary factors (family history of oral cancer), mechanical factors (chronic oral trauma or irritation), metaphysical factors (enemy and spiritual attacks), therapeutic factors (fluoride use, stem‐cell transplantation, and dental amalgam filling), local factors (dental caries, prior history of oral cancer, poor oral hygiene or health, oral premalignant lesions) and systemic factors (immunosuppression, autoimmune diseases, stress, older age, and biological sex). Overall, these factors could be grouped into established and probable factors. The established factors are tobacco use, alcohol use, betel quid/areca nut use, and human papillomavirus infection while the other factors are the probable ones [107].

However, in this review, only those findings on the prevalence and determinants of awareness/knowledge on the established risk/aetiological factors of oral cancer were focused on. The prevalence of knowledge of smoked/smokeless tobacco and tobacco products as an oral cancer risk/aetiological factor in the reviewed contemporary articles had a wide range; it ranged from 1.1% among a sample of secondary school students in Nigeria to 99.7% among a sample of homeopathy and ayurveda students in India [74, 95]. Similar wide range was also found concerning alcohol use, with the reported prevalence of knowledge ranging from 5.6% among a sample of medical students in Iraq to ≈100% among a sample of dentists in the Democratic Republic of Congo [93, 101]. Also, for human papillomavirus infection, the reported prevalence of knowledge ranged from 1.9% among a sample of patients in India to 90.3% among a sample of dental students in Malaysia [44, 50]. Lastly, for betel quid/areca nut use, the reported prevalence of knowledge ranged from 2.7% among dentists in Nigeria to 66% among community dwellers in India [22, 99]. Overall, the observed disparities in the knowledge of oral cancer risk/aetiological factor reveals wide inequalities in the knowledge of the disease, with low prevalence reported among lay populations and high among healthcare trainees and professionals; this therefore necessitates the need for a more robust and tailored approach on public health education on oral cancer, with a huge focus on the lay public.

Notably, only very few (three articles in total) of the reviewed contemporary articles reported the determinants of awareness/knowledge on the established risk/aetiological factors of oral cancer [21, 52, 86]. This observation indicates that most of these studies lacked robust statistical analysis (such as odds ratios) [67]. In those few studies, age [21], gender [52, 86], level of education [86], sexuality [52], family history of oral cancer [21, 86], history of alcohol use [52], history of sexual activity [52], and intent to receive vaccination against human papillomavirus infection [52] were found to be statistically significant determinants of knowledge of the risk/aetiological factors of oral cancer. On the other hand, only history of sexually transmitted infections was the only determinant that was found to be a nonsignificant determinant of such knowledge. Based on the above, it can be summarised that demographic factors, behavioural factors, and medical status determines a person's status concerning the acquisition of such knowledge on oral cancer. However, to ensure equitable access to adequate knowledge of the risk or aetiological factors of oral cancer, it is therefore recommended that public health policies and interventions are favourably focused on those sociodemographic groups that are less likely to have such knowledge.

### 2.4 Current State of Awareness and Knowledge on the Clinical Features of Oral Cancer

The understanding of the current state of global awareness and knowledge on the clinical features of oral cancer is very crucial, as this provides insight on what the public knows about the signs and symptoms of the disease, as this will help in identifying the causes of the problem of delayed oral cancer presentation across the world populations. The findings reported in this section were based on thirty‐three contemporary peer‐reviewed articles which investigated public awareness and knowledge of the clinical features of oral cancer (Table S4) [7, 19, 21, 22, 24, 27, 28, 30, 31, 32, 44, 46, 48, 49, 50, 51, 53, 57, 58, 62, 69, 70, 71, 72, 75, 82, 83, 84, 86, 91, 92, 95, 108].

The analysis of the geographical distribution of the populations investigated in these reviewed articles showed that the participants were predominantly in Asia and Europe, while only few to none were in Africa, Australia, North America, and South America. This therefore reveals the need for further empirical studies investigating the knowledge of the clinical features of oral cancer in these identified continents with low empirical evidence on this topic area. Furthermore, the populations investigated were predominantly patients, community dwellers, and students while only a small proportion of them were health professionals (dentists, medical doctors, nurses, and healthcare assistants) and military personnel. This analysis further shows that the existing contemporary empirical evidence had overwhelmingly focused on patients, community dwellers, and students, with little to no interest on populations outside these three groups. Hence, there is a need for further research investigating other unexplored and at‐risk groups such as commercial sex workers and farmers [63, 64].

Notably, several clinical features of oral cancer were known to the participants in these reviewed contemporary articles. These features include white oral patch, red oral patch, red‐white oral patch, nonhealing oral ulcer/sore, oral numbness, oral/neck lump/mass, oral necrosis, chronic oral infection, dysphagia, difficulty in chewing, difficulty in mouth opening, xerostomia, toothache, lymphadenopathy, change in voice, dental caries, oral abscess, and pain. However, in this review, only the common oral cancer clinical features—pain, chronic non‐healing oral ulcer, pathologic tooth mobility, oral lump, and oral bleeding—were focused on.

The prevalence of awareness/knowledge of pain as a clinical feature of oral cancer was not elaborate in the reviewed articles; however, those few articles reporting its prevalence reported a range of 0.98% among a sample of dental students and dentists in Nepal to 47.2% among community dwellers in Saudi Arabia [30, 75]. The low prevalence of awareness of pain among dental students and dentists in Nepal [75] is concerning, this is because they are oral healthcare trainees and professionals who are supposed to be more knowledgeable, compared to the lay populations, about oral cancer clinical features. From the review of the methods adopted in the Nepal study in order to identify the probable causes of the low prevalence of knowledge among its participants, it was observed that questionnaire used for its data collection could, probably, have used an open‐ended question to enquire about oral cancer clinical features from its participants; and this could have been the reason why the prevalence rate on awareness/knowledge of pain was low in the study [75]. Nonetheless, this finding as well as the overall findings—a prevalence of 0.98% to 47.2%—indicates that knowledge/awareness of pain as an oral cancer clinical feature is generally low across all populations studied in recent years.

However, the range of the prevalence of awareness/knowledge of the other common clinical feature of oral cancers—chronic non‐healing oral ulcer, pathologic tooth mobility, oral lump, and oral bleeding—were found to be higher than that of pain. The reported prevalence of awareness/knowledge of chronic non‐healing oral ulcer ranged from 20.3% among a sample of patients in India to 90.3% among a sample of patients in Australia [21, 50]. The prevalence of awareness/knowledge of pathological tooth mobility ranged from 3.54% among a sample of dental students and dentists in Nepal to 59.67% among a sample of community dwellers in Jordan [57, 75]. The prevalence of awareness/knowledge of oral lump ranged from 5.1% among a sample of community dwellers in India to a sample of clinical staff in the United Kingdom [48, 82]. The prevalence of awareness/knowledge of oral bleeding ranged from 1.96% among a sample of dental students and dentists in Nepal to 74.97% among a sample of community dwellers in Jordan [57, 75]. As earlier identified, there is a plausible need to interpret the study by Poudel et al. [75] with extreme caution considering its methodological limitations caused by poor questionnaire calibration which was prone to recall bias [109]. Overall, the disparities observed in the prevalence of awareness/knowledge of each of the common clinical feature of oral cancers was very wide. This shows that substantial inequalities exist globally on the awareness/knowledge of the signs and symptoms of oral cancer; hence, this calls for the need to rejuvenate public health education programmes on oral cancer globally.

Pertinently, only very few of the reviewed contemporary articles reported the determinants of awareness/knowledge of the clinical features of oral cancer [21, 27, 86]. In these articles, age, level of income, level of education, family history of oral cancer, place of residence, and frequency of mouth self‐examination were found to be significant determinants of such awareness/knowledge [21, 27, 86]. Specifically, those with higher age, higher level of income, higher level of education, positive family history of oral cancer, residential location in more urbanised locations, and higher frequency of mouth self‐examination practices were more likely to be aware/knowledgeable about the clinical features of oral cancer when compared with those outside these sociodemographic groups.

### 2.5 Current State of Awareness and Knowledge on the Prevention of Oral Cancer

The understanding of the current state of global awareness and knowledge on the prevention of oral cancer is very crucial, as this provides insight on what the public knows about ways to prevent the disease from occurring in the first instance, as this will help in enhancing reducing the incidence of oral cancer across the world populations. The findings reported in this section were based on seventeen contemporary peer‐reviewed articles which investigated public awareness and knowledge on the prevention of oral cancer (Table S5) [24, 26, 27, 28, 30, 44, 49, 53, 70, 71, 72, 87, 96, 98, 102, 110, 111].

Based on the analysis of the populations studied in these reviewed contemporary articles, it can be concluded that most of the evidence were focused on Asian populations while only very scanty evidence is available on North America and Europe, and no known evidence was on African, Latin American, and the Australian and Oceanian populations. Also, all these studied populations were patients, community dwellers, health science students, and dentists; none of them specifically investigated oral cancer at‐risk populations like commercial sex workers, industrial workers, and farmers [63, 64]. This paucity of contemporary evidence identifies the need for future research investigating this topic areas, especially among those populations where contemporary evidence is lacking.

From the findings reported in the reviewed articles, it was identified that most of these articles reported findings on the awareness on the preventability of oral cancer while only few reported findings on the awareness/knowledge of the ways in which oral cancer can be prevented. Concerning the awareness on the preventability of oral cancer, the reported prevalence ranged from 16.67% among a sample of community dwellers in India to 92.7% among a sample of dental students in Malaysia [44, 49].

However, regarding prevention, some articles reported overall awareness of strategies, while others examined specific approaches. In those articles reporting the overall awareness on oral cancer preventative strategies, the range was from 27.3% among a sample of dental students in India to 30.7% among a sample of dental students in Nepal [70, 71].

In those articles reporting the prevalence rates on the awareness/knowledge of specific preventative strategies against oral cancer, only the following strategies were reported: oral cancer self‐examination [24, 72, 110]; engagement in sporting activities [53]; cessation/avoidance of smoking [53, 87]; cessation/avoidance of alcohol use [87]; consumption of vegetables [53]; oral cancer screening [26, 28]; and vaccination against human papillomavirus infection [98].

Only oral cancer self‐examination, cessation/avoidance of smoking, and oral cancer screening were the preventative strategies that were reported in multiple studies. For oral cancer self‐examination strategy, the prevalence of its awareness/knowledge ranged from 11.68% among a sample of community dwellers and patients in India to 55.2% among a sample of community dwellers in Malaysia [24, 72]. For cessation/avoidance of smoking strategy, the prevalence of its awareness/knowledge ranged from 68.1% among a sample of dental students and dentists in Palestine to 80.2% among a sample of patients in Saudi Arabia [53, 87]. For oral cancer screening, the prevalence of its awareness/knowledge ranged from 61.4% among a sample of community dwellers in China to 72.7% among a sample of community dwellers in Saudi Arabia [26, 28]. However, for those preventative strategies that were reported in only one study—engagement in sporting activities, consumption of vegetables, cessation/avoidance of alcohol use, and vaccination against human papillomavirus infection—the prevalence of their awareness/knowledge rate were reported to be 40.1% (among a sample of patients in Saudi Arabia), 83.8% (among a sample of patients in Saudi Arabia), 62.3% (among a sample of dental students and dentists in Palestine), and 61.5% (among a sample of health science students in India), respectively [53, 87, 98].

Overall, substantial disparities exist in the global awareness and knowledge on the prevention on oral cancer, and notably, none of these studies reported the determinants of these knowledge. These observed disparities coupled with the current lack of evidence on the determinants of these awareness/knowledge on oral cancer calls for the need for further research on this topic area.

### 2.6 Current State of Awareness and Knowledge on the Diagnosis of Oral Cancer

The understanding of the current state of global awareness and knowledge on the diagnosis of oral cancer is very crucial, as this provides insight on what the public knows about how the disease is diagnosed, as this will help in enhancing early clinical presentation of people afflicted with the disease [112]. The findings reported in this sub‐section were based on eight contemporary peer‐reviewed articles which investigated public awareness and knowledge on the diagnosis of oral cancer (Table S6) [29, 44, 45, 57, 71, 75, 76, 77].

An analysis of the geographical and sociodemographic distributions of the populations investigated in these eight articles revealed that all these articles were focused on Asian populations, all of whom were either medical students, dental students, or dentists. Unfortunately, none of these articles reported findings on lay populations. Having contemporary evidence on the awareness and knowledge on the diagnosis of oral cancer, particularly among lay populations is very important for public health strategy on oral cancer prevention, as research evidence has shown that people with inadequate knowledge or misconceptions on disease diagnosis (or diagnostic techniques) tend to clinically present late for treatment due to reasons associated with fears and ignorance [112, 113, 114].

Notably, in some of the reviewed articles, only general awareness on oral cancer diagnostic techniques were reported while in other articles, the specific knowledge of these techniques was reported. Concerning general awareness on oral cancer diagnostic techniques, its prevalence was found to be 31.92% among a sample of medical students in India [44], between 8.9% and 74.1% among samples of dental students in India, Nepal, and Malaysia [44, 45, 71, 75], and between 33.3% and 59.4% among samples of dentists in Nepal [75, 77].

In those articles reporting specific knowledge on oral cancer diagnostic techniques, the following techniques were identified by their study participants: biopsy [29, 77]; the use of toluidine blue [77]; barium swallow [57]; panoramic radiographs [77]; endoscopy [77]; blood tests [77]; computed tomography scan [77]; magnetic resonance imaging [77]; and oral brush biopsy [77]. Notably, the gold standard technique for oral cancer diagnosis is biopsy, and the prevalence of its knowledge was reported in two articles, ranging from 3.0% among a sample of dentists in Nepal to > 79% among a sample of dental students in Saudi Arabia [29, 77].

However, for the other identified techniques—the use of toluidine blue, barium swallow, panoramic radiographs, endoscopy, blood tests, computed tomography scan, magnetic resonance imaging, and oral brush biopsy—the prevalence of their knowledge were 3.0%, 27.54%, 35.19%, 59.67%, 66.56%, 60.55%, 72.68%, and 75.08%, respectively [57, 77]. Notably, the prevalence rates for all these other techniques were reported among a sample of community dwellers in Jordan [57], except for toluidine blue which was reported among a sample of dentists in Nepal [77].

Overall, none of these reviewed contemporary articles reported the determinants of the awareness/knowledge on the diagnosis of oral cancer. This lack of contemporary evidence on the determinants of this knowledge among global populations is a huge gap that needs to be filled. This gap underscores the importance of new empirical studies on oral cancer diagnosis.

### 2.7 Current State of Awareness and Knowledge on the Treatment of Oral Cancer

The understanding of the current state of global awareness and knowledge on the treatment of oral cancer is very crucial, as this provides insight on what the public knows about how the disease is treated, as this will help in enhancing adequate management of people afflicted with the disease [112]. The findings reported in this section were based on thirteen contemporary peer‐reviewed articles which investigated public awareness and knowledge on the treatment of oral cancer (Table S7) [19, 23, 27, 28, 30, 45, 49, 51, 58, 61, 69, 82, 83].

An analysis of the geographical and sociodemographic distributions of the populations investigated in these thirteen articles revealed that these articles were predominantly focused on Asian populations, with very few of them on African and European populations. Also, the surveyed populations were focused on patients, clinical staff (medical doctors, nurses, and healthcare assistants), community dwellers, medical and dental students, dentists, healthy relatives of cancer patients, and military personnels. Overall, this population distributions show that there exist huge paucities of contemporary evidence on the global awareness and knowledge on oral cancer treatment, as some peculiarly at‐risk populations (such as farmers, commercial sex workers, and industrial workers) were not particularly investigated in the reviewed studies [63, 64]. This therefore calls for further empirical investigations on at‐risk populations and among populations with little or no evidence—such as the African, American, European, and Australian and Oceanian populations—as such evidence is needed for robust planning of tailored and effective public health education interventions on oral cancer.

Notably, some of the reviewed articles reported findings on their participants' awareness of the treatability of oral cancer, some reported findings on their participants' specific knowledge of oral cancer treatment options, and some reported findings on their participants' specific knowledge of the experts that treat oral cancer. Concerning the knowledge on the treatability of oral cancer, its prevalence ranged from 30.56% among a sample of community dwellers in India to 90.6% among a sample of dental students in Malaysia [45, 49].

Concerning the knowledge of oral cancer treatment options, its prevalence among the populations surveyed was (or ranged between) 37.5%–59.8% for chemotherapy, 56.3% for radiotherapy, 16.67%–55.0% for surgery, 5.56%–37.25% for both chemotherapy and surgery, and 70% for chemotherapy, radiotherapy, and surgery [23, 28, 49, 51]. Notably, these findings on the knowledge of oral cancer treatment options were based on data obtained from samples of community dwellers in India and Saudi Arabia, and patients in India only, as no relevant data was found on populations elsewhere.

Concerning the knowledge of the experts that treat oral cancer, its prevalence among the populations surveyed was (or ranged between) 9.6%–100% for oral and maxillofacial surgeons, 67.1%–98% for oncologists, 21.3% for otorhinolaryngologists, and 2% for dentists [82, 83]. Notably, these findings on the knowledge of the experts that treat oral cancer were based on data obtained from samples of clinical staff (medical doctors, nurses, and healthcare assistants) in the United Kingdom and medical students in Syria only, as no relevant data was found on populations elsewhere. Also, the findings from these articles revealed inadequate knowledge of the entire oral cancer treatment team, as nurses, prosthodontists, and psychologists were not identified as part of these experts [115, 116, 117].

Overall, none of the reviewed articles reported any findings on the determinants of knowledge on oral cancer treatment. However, the knowledge of these determinants is crucial for the in‐depth and broad understanding of the factors that influences and individual status of knowledge on this topic area on oral cancer, as this knowledge will help in the planning of the scope of the public health education interventions in those populations affected. These observations show the need to explore determinants of treatment knowledge through targeted empirical studies.

## 3 Summary of the Identified Gaps and Future Directions

This narrative review highlights several important gaps in the existing evidence on oral cancer awareness and knowledge. Although research activity has increased in recent years, studies remain concentrated in specific regions. Most of the available evidence comes from Asian countries, while Africa, Latin America, North America and Oceania remain underrepresented, which restricts the global relevance and comparability of current findings [1, 24, 56, 104]. This regional imbalance mirrors broader public health concerns about the need for more geographically inclusive data to support effective oral cancer prevention strategies [66, 112]. A second gap relates to population coverage. Many studies focus on clinical or educated populations and overlook vulnerable groups such as industrial workers, farmers, commercial sex workers and individuals with low health literacy, even though these groups often face higher exposure to risk factors [24, 49, 51, 118]. Their exclusion limits understanding of awareness patterns across diverse communities.

A further gap concerns the depth of analysis. While numerous surveys report awareness or knowledge levels, fewer explore the determinants that shape these outcomes. Only a limited number of studies use multivariate or theory‐informed analytical approaches to investigate how sociodemographic, behavioural or contextual characteristics influence awareness, help‐seeking or early detection behaviour [26, 44, 47]. Previous methodological research has emphasised that deeper analytical approaches are critical for designing targeted and equitable health interventions [109, 113].

To advance the field, future work should prioritise expanding research into underrepresented regions and strengthening collaboration through global funding entities such as WHO, NIH and RSTMH, as well as established capacity‐building networks including the STEPwise Surveillance system and the Global Burden of Disease Collaborative Network [107, 119]. Improving representation also requires intentional engagement with vulnerable and high‐risk populations through community‐based recruitment, culturally tailored awareness activities and outreach strategies such as mobile screening and peer‐led education [51, 65, 66, 105].

Future research would further benefit from the adoption of validated tools, longitudinal designs and advanced analytical models to better identify the determinants of awareness and knowledge [67, 68, 119]. Although meta‐analysis may be valuable, its feasibility will depend on reducing the substantial heterogeneity observed across existing studies in measurement tools, outcome definitions and reporting practices [67, 68]. Standardisation in future research will help create a stronger foundation for meaningful quantitative synthesis.

## 4 Conclusion

This review provides a broader understanding on empirical evidence on the current state of global awareness and knowledge on oral cancer—information sources, definitions, risk factors, clinical features, prevention, diagnosis and treatment—by highlighting several evidence gaps on global oral cancer research. By implementing these recommended future directions, global public health systems can move toward a more equitable and comprehensive understanding of oral cancer awareness and knowledge, ultimately strengthening prevention and early detection efforts on a global scale.

## Author Contributions

**Kehinde Kazeem Kanmodi:** conceptualization, investigation, funding acquisition, writing – original draft, methodology, validation, visualization, software, formal analysis, project administration, data curation, resources. **Yovanthi Anurangi Jayasinghe:** writing – original draft, writing – review and editing. **Ruwan Duminda Jayasinghe:** supervision, writing – review and editing. **Emeka Benjamin Okeke:** writing – original draft, writing – review and editing. **Misheck Julian Nkhata:** supervision, writing – review and editing. **Lawrence Achilles Nnyanzi:** supervision, writing – review and editing.

## Ethics Statement

The authors have nothing to report.

## Conflicts of Interest

Kehinde Kazeem Kanmodi is an Editorial Board member of *Health Science Reports* and a co‐author of this article. To minimize bias, they were excluded from all editorial decision‐making related to the acceptance of this article for publication. Other authors have no conflict of interest to declare.

## Transparency Statement

The lead author Kehinde Kazeem Kanmodi affirms that this manuscript is an honest, accurate, and transparent account of the study being reported; that no important aspects of the study have been omitted; and that any discrepancies from the study as planned (and, if relevant, registered) have been explained.

## Supporting information


**Table S1:** Summary of current primary studies reporting the sources of information on oral cancer among diverse population groups. **Table S2:** Summary of current primary studies reporting the prevalence and determinants of awareness and knowledge of the term “oral cancer” (or its subtype) among diverse population groups. **Table S3:** Summary of the included primary studies which investigated knowledge of the risk or aetiological factors of oral cancer among its study participants. **Table S4:** Summary of current primary studies reporting the prevalence and determinants of awareness and knowledge of the clinical features of oral cancer among diverse population groups. **Table S5:** Summary of the included primary studies which investigated the prevalence and determinants of knowledge of the preventability and preventative strategies of oral cancer among their study participants. **Table S6:** Summary of current primary studies reporting the prevalence and determinants of awareness and knowledge on oral cancer diagnostic approaches. **Table S7:** Summary of current primary studies reporting the prevalence and determinants of awareness and knowledge on the treatability and treatment options of oral cancer.

## Data Availability

The authors confirm that the data supporting the findings of this study are available within the article and its Supporting Information.

## References

[1] A. Chamoli , A. S. Gosavi , U. P. Shirwadkar , et al., “Overview of Oral Cavity Squamous Cell Carcinoma: Risk Factors, Mechanisms, and Diagnostics,” Oral Oncology 121 (2021): 105451, 10.1016/j.oraloncology.2021.105451. 34329869

[2] D. Lenoci , E. Moresco , S. Cavalieri , et al., “Oral Cancer in Young Adults: Incidence, Risk Factors, Prognosis, and Molecular Biomarkers,” Frontiers in Oncology 14 (2024): 1452909, 10.3389/fonc.2024.1452909. 39421447 PMC11484398

[3] F. Bray , M. Laversanne , H. Sung , et al., “Global Cancer Statistics 2022: GLOBOCAN Estimates of Incidence and Mortality Worldwide for 36 Cancers in 185 Countries,” CA: A Cancer Journal for Clinicians 74, no. 3 (2024): 229–263, 10.3322/caac.21834. 38572751

[4] K. Senevirathna , Y. A. Jayasinghe , S. M. Jayawickrama , H. Amarasinghe , and R. D. Jayasinghe , “Oral Cancer Disease Among the Poor: A Sri Lankan Context,” Oral 3, no. 3 (2023): 420–436, 10.3390/oral3030034.

[5] J. Wu , H. Chen , Y. Liu , R. Yang , and N. An , “The Global, Regional, and National Burden of Oral Cancer, 1990–2021: A Systematic Analysis for the Global Burden of Disease Study 2021,” Journal of Cancer Research and Clinical Oncology 151, no. 2 (2025): 53, 10.1007/s00432-025-06098-w. 39875744 PMC11775039

[6] A. K. Jain , “Oral Cancer Screening: Insights Into Epidemiology, Risk Factors, and Screening Programs for Improved Early Detection,” Cancer Screening and Prevention 3, no. 2 (2024): 97–105, 10.14218/CSP.2023.00029S.

[7] Y. de Lima Medeiros , G. de Matos Silveira , V. B. Clemente , I. C. G. Leite , E. M. Vilela , and L. D. de Abreu Guimarães , “Knowledge About Oral Cancer Among Dental Students and Primary Health Care Dentists: A Brazilian Study,” Journal of Dental Education 86, no. 11 (2022): 1488–1497, 10.1002/jdd.13021. 35851666

[8] M. Á. González‐Moles , M. Aguilar‐Ruiz , and P. Ramos‐García , “Challenges in the Early Diagnosis of Oral Cancer, Evidence Gaps and Strategies for Improvement: A Scoping Review of Systematic Reviews,” Cancers 14, no. 19 (2022): 4967, 10.3390/cancers14194967. 36230890 PMC9562013

[9] R. Mauceri , M. Bazzano , M. Coppini , P. Tozzo , V. Panzarella , and G. Campisi , “Diagnostic Delay of Oral Squamous Cell Carcinoma and the Fear of Diagnosis: A Scoping Review,” Frontiers in Psychology 13 (2022): 1009080, 10.3389/fpsyg.2022.1009080. 36405204 PMC9669962

[10] N. A. Ahuja , S. K. Kedia , K. D. Ward , et al., “Effectiveness of Interventions to Improve Oral Cancer Knowledge: A Systematic Review,” Journal of Cancer Education 37, no. 3 (2022): 479–498, 10.1007/s13187-021-01963-x. 33506408

[11] K. Kanmodi , P. Kanmodi , M. Ogbeide , and J. Nwafor , “Head and Neck Cancer Literacy in Nigeria: A Systematic Review of the Literature,” Annals of Public Health Issues 1, no. 1 (2021): 25–49, 10.2478/aphi-2021-0004.

[12] S. de Mattos Camargo Grossmann , A. C. R. Sales , D. S. Reis , et al., “Knowledge of Oral Cancer by a Brazilian Population,” Journal of Cancer Education 36, no. 5 (2021): 965–970, 10.1007/s13187-020-01722-4. 32124247

[13] P. A. Pullon and A. S. Miller , “Oral Cancer Knowledge: Results of a Survey of Pennsylvania Dentists in 1971,” Journal of the American Dental Association 86, no. 1 (1973): 149–152, 10.1016/S0002-8177(73)61037-9. 4508873

[14] D. Sadowsky , C. Kunzel , and J. Phelan , “Dentists' Knowledge, Case‐Finding Behavior, and Confirmed Diagnosis of Oral Cancer,” Journal of Cancer Education 3, no. 2 (1988): 127–134, 10.1080/08858198809527926. 3275229

[15] J. Amzat , O. Razum , and K. K. Kanmodi , “Polio‐Philanthropy in Africa: A Narrative Review,” Health Science Reports 6, no. 6 (2023): e1339, 10.1002/hsr2.1339. 37324246 PMC10265140

[16] A. A. Salami , K. K. Kanmodi , and J. Amzat , “The Roles of Chaplains in Dispelling Cancer Myths in Nigeria: A Narrative Review,” Health Science Reports 6, no. 8 (2023): e1502, 10.1002/hsr2.1502. 37614282 PMC10442495

[17] M. H. A. Banna , S. Kundu , and S. M. Y. Arafat , “Eating Disorders in Bangladesh: A Narrative Review,” Health Science Reports 8, no. 3 (2025): e70537, 10.1002/hsr2.70537. 40060296 PMC11885170

[18] M. Nakhaie , M. R. Z. Rukerd , N. Farsiu , et al., “Mpox and Viral Co‐Infections: A Narrative Review,” Health Science Reports 8, no. 2 (2025): e70464, 10.1002/hsr2.70464. 40012816 PMC11861034

[19] E. D. Yalcin and H. Gundogar , “Knowledge and Awareness About Oral Cancer Among Dental Patients in Southeastern Anatolia,” Annals of Medical Research 27, no. 2 (2020): 616–622.

[20] D. Ozdemir‐Ozenen , O. Tanriover , G. Ozenen , M. Ozdemir‐Karatas , C. Ozcakir‐Tomruk , and J. Tanalp , “Dental Education for Prevention of Oral Cancer in Turkey: Needs for Changing the Curriculum,” Journal of Cancer Education 37, no. 5 (2022): 1496–1503, 10.1007/s13187-021-01989-1. 33742374

[21] J. J. Zachar , B. Huang , and E. Yates , “Awareness and Knowledge of Oral Cancer Amongst Adult Dental Patients Attending Regional University Clinics in New South Wales, Australia: A Questionnaire‐Based Study,” International Dental Journal 70, no. 2 (2020): 93–99, 10.1111/idj.12533. 31743437 PMC9379200

[22] K. Oswal , R. Kanodia , A. Pradhan , et al., “Assessment of Knowledge and Screening in Oral, Breast, and Cervical Cancer in the Population of the Northeast Region of India,” JCO Global Oncology 6 (2020): 601–609, 10.1200/JGO.19.00257. 32302235 PMC7193798

[23] M. Deshpande , M. Meshram , P. Paul , et al., “Assessment of Cancer Patients' Relatives' Knowledge, Perception, and Attitude Toward Cancer,” Cureus 15, no. 8 (2023): e43457, 10.7759/cureus.43457. 37711954 PMC10498802

[24] S. Shahabudin , N. Mohd Shariff , H. Roslan , H. Ahmad Yusof , and R. Hami , “Oral Cancer Awareness and Knowledge Among Marginalised Group in Sungai Petani, Kedah, Malaysia,” Archives of Orofacial Sciences 15, no. 1 (2020): 11–21.

[25] E. A. S. D. Somathunga , D. M. S. H. Dissanayaka , D. R. D. L. Ratnayake , and R. D. Jayasinghe , “Awareness of Oral Cancer and OPMDs Among Patients Attending the University Dental Hospital, Peradeniya, Sri Lanka,” Asian Pacific Journal of Cancer Care 6, no. 1 (2021): 47–51.

[26] J. Adeoye , C. S. Chu , S. W. Choi , and P. Thomson , “Oral Cancer Awareness and Individuals' Inclination to Its Screening and Risk Prediction in Hong Kong,” Journal of Cancer Education 37, no. 2 (2022): 439–448, 10.1007/s13187-020-01834-x. 32705524

[27] X. H. Zhou , Y. Huang , C. Yuan , et al., “A Survey of the Awareness and Knowledge of Oral Cancer Among Residents in Beijing,” BMC Oral Health 22, no. 1 (2022): 367, 10.1186/s12903-022-02398-6. 36031600 PMC9420274

[28] M. Al Hulaibi , A. Alhazemi , A. Alshamakhi , et al., “The Association Between Sociodemographic Characteristics and Knowledge About Oral Cancer Among Jazan Population, Saudi Arabia,” Journal of Family Medicine and Primary Care 11, no. 9 (2022): 5581–5587, 10.4103/jfmpc.jfmpc_595_22. PMC9730950 36505661

[29] B. Tarakji , “Knowledge, Awareness, and Attitude Among Dental Students Regarding Oral Cancer in Saudi Arabia,” Annals of African Medicine 21, no. 4 (2022): 444–450, 10.4103/aam.aam_185_21. 36412349 PMC9850895

[30] F. H. Dallak , F. A. Alharbi , A. H. Alhazmi , et al., “Community Awareness Regarding Smokeless Tobacco (Shamma) as a Cause of Oral Cancer in Jazan Region, Saudi Arabia: A Cross‐Sectional Study,” Journal of Family Medicine and Primary Care 13, no. 11 (2024): 4885–4893, 10.4103/jfmpc.jfmpc_534_24. PMC11668448 39722995

[31] H. Gerber , T. Gedrange , P. Szymor , et al., “Oral Cancer Awareness Among Patients at 3 University Hospitals in Poland and Germany: A Survey Research,” Advances in Clinical and Experimental Medicine 31, no. 6 (2022): 607–613, 10.17219/acem/146455. 35195963 PMC13539080

[32] N. Prado , R. Bonan , A. Leonel , et al., “Awareness on Oral Cancer Among Patients Attending Dental School Clinics in Brazil,” Medicina Oral Patología Oral y Cirugia Bucal 25, no. 1 (2020): e89–e95, 10.4317/medoral.23207. 31880286 PMC6982986

[33] K. Rupel , M. Biasotto , M. Gobbo , et al., “Knowledge and Awareness of Oral Cancer: A Cross‐Sectional Survey in Trieste, Italy,” Frontiers in Oral Health 4 (2023): 1056900, 10.3389/froh.2023.1056900. 36794079 PMC9922703

[34] S. M. Razavi , B. Tahani , L. Maleki , and D. B. N. Esfahani , “Oral Cancer Knowledge Among Dental Patients in Isfahan,” Dental Research Journal 21 (2024): 2. 38425322 PMC10899157

[35] J. N. Nwafor , K. K. Kanmodi , and B. A. Amoo , “How Enlightening and Reliable Are Cancer‐Related Posts on Social Media Platforms? Opinions of a Sample of Nigerians,” Journal of Health and Allied Sciences NU 11, no. 03 (2021): 141–146.

[36] K. Kanmodi , O. Abolade , J. Amzat , and L. Nnyanzi , “Analysis of Global Search and Research Interests on Dentists Using Infoveillance and Bibliometric Approaches,” Oral 3, no. 1 (2022): 11–30.

[37] K. K. Kanmodi , A. A. Salami , J. N. Nwafor , C. A. Olomo , and L. A. Nnyanzi , “Trend Analysis of Global Web Searches (2004–2022) on Oral Cancer and Its Major Risk Factors,” Journal of Health and Allied Sciences NU 13, no. 03 (2023): 373–379.

[38] Y. A. Jayasinghe , K. K. Kanmodi , R. M. Jayasinghe , and R. D. Jayasinghe , “Assessment of Patterns and Related Factors in Using Social Media Platforms to Access Health and Oral Health Information Among Sri Lankan Adults, With Special Emphasis on Promoting Oral Health Awareness,” BMC Public Health 24, no. 1 (2024): 1472, 10.1186/s12889-024-19008-5. 38824505 PMC11143610

[39] K. K. Kanmodi , A. A. Salami , and J. N. Nwafor , “mHealth Interventions to Improve Public Knowledge of HPV‐Associated Oropharyngeal Cancer in the UK,” Exploration of Digital Health Technologies 2, no. 5 (2024): 271–278.

[40] K. K. Kanmodi , Y. A. Jayasinghe , R. D. Jayasinghe , et al., “The Understanding of Digital Communication Experts and Oral Cancer At‐Risk Persons on Oral Cancer, Their Uptake of Educational Mobile Health Applications on Oral Cancer, and Their Opinions on How a Good Application of Such Should Look Like: Findings From a Qualitative Study,” BMC Oral Health 25, no. 1 (2025): 224, 10.1186/s12903-025-05614-1. 39939967 PMC11823197

[41] N. Maniyar , G. S. Sarode , S. C. Sarode , and S. Thakkar , “ChatGPT Conversations on Oral Cancer: Unveiling Chatgpt's Potential and Pitfalls,” Oral Oncology Reports 10 (2024): 100280.

[42] J. E. Gallagher and L. Hutchinson , “Analysis of Human Resources for Oral Health Globally: Inequitable Distribution,” International Dental Journal 68, no. 3 (2018): 183–189. 29297930 10.1111/idj.12349 PMC9378901

[43] M. Han and E. Lee , “Effectiveness of Mobile Health Application Use to Improve Health Behavior Changes: A Systematic Review of Randomized Controlled Trials,” Healthcare Informatics Research 24, no. 3 (2018): 207–226, 10.4258/hir.2018.24.3.207. 30109154 PMC6085201

[44] S. Gunjal , D. G. S. Pateel , R. Z. S. Lim , L. L. Yong , and H. Z. Wong , “Assessing Oral Cancer Awareness Among Dental and Medical Students of a Malaysian Private University,” International Dental Journal 70, no. 1 (2020): 62–69, 10.1111/idj.12524. 31691268 PMC9379211

[45] Z. W. Chan , Y. F. Phuan , P. Y. Ooi , et al., “An Assessment of Oral Cancer Knowledge, Attitudes, and Practices Among Undergraduate Students in Malaysian Dental Schools,” BMC Oral Health 23, no. 1 (2023): 617, 10.1186/s12903-023-03354-8. 37653402 PMC10469815

[46] P. Bhat , S. Sushma , M. Jayachandra , C. Aruna , and M. Murthy , “Awareness About Oral Cancer Among Nonhealth Professional Students—A Cross‐Sectional Study in Bengaluru City,” Journal of Oral and Maxillofacial Pathology 24, no. 3 (2020): 492–498, 10.4103/jomfp.jomfp_304_20. 33967486 PMC8083434

[47] V. Kadashetti , K. Shivakumar , M. Choudhary , S. Patil , and M. Gawnde , “Awareness and Knowledge of Tobacco Associated Risk of Development of Oral Cancer and Oral Potentially Malignant Disorders Among Patients Visiting a Dental College,” Journal of Family Medicine and Primary Care 9, no. 5 (2020): 2244–2247, 10.4103/jfmpc.jfmpc_181_20. PMC7380825 32754481

[48] S. Singh , A. Alok , L. Nagesh , K. K. Shivalingesh , V. K. Sah , and I. D. Singh , “Awareness of Oral Cancer, Oral Premalignant Disorders and Their Risk Factors Among Adult Population in Bareilly City,” Indian Journal of Dental Sciences 12, no. 3 (2020): 126–131.

[49] S. Muthanandam , B. V. Babu , J. Muthu , R. Suganya , N. Vezhavendhan , and M. Kishore , “Assessment of Knowledge, Awareness and Attitude Towards Oral Precancer and Cancer Among Narikuravar Population in Pondicherry State,” South Asian Journal of Cancer 10, no. 4 (2021): 225–229, 10.1055/s-0041-1733316. 34984200 PMC8719971

[50] P. Anirudh , S. Ty , S. A , and R. S , “Assessment of Knowledge of and Attitude Toward Oral Cancer Among the Outpatient Population in a Tertiary Care Rural Hospital,” Cureus 15, no. 3 (2023): e36637, 10.7759/cureus.36637. 37155442 PMC10122915

[51] A. Chugh , A. Kaur , P. Bhardwaj , et al., “Gap Areas in Mitigation of Oral Cancer: A Cross‐Sectional Study Evaluating Awareness and Knowledge of Risk Factors in Oral Cancer in a Tertiary Hospital,” National Journal of Maxillofacial Surgery 14, no. 1 (2023): 27–34, 10.4103/njms.njms_427_21. 37273436 PMC10235728

[52] R. H. Dodd , M. Freeman , F. Dekaj , et al., “Awareness of the Link Between Human Papillomavirus and Oral Cancer in UK University Students,” Preventive Medicine 150 (2021): 106660, 10.1016/j.ypmed.2021.106660. 34081936

[53] M. Jafer , R. Crutzen , A. Ibrahim , et al., “Using the Exploratory Sequential Mixed Methods Design to Investigate Dental Patients' Perceptions and Needs Concerning Oral Cancer Information, Examination, Prevention and Behavior,” International Journal of Environmental Research and Public Health 18, no. 14 (2021): 7562, 10.3390/ijerph18147562. 34300012 PMC8307210

[54] A. Alsalhani , B. Tarakji , F. Mehsen Alali , et al., “Knowledge and Awareness of Dental Students Regarding Human Papillomavirus and Oral Cancer in Saudi Arabia,” Asian Pacific Journal of Cancer Prevention 25, no. 11 (2024): 3927–3934, 10.31557/APJCP.2024.25.11.3927. 39611917 PMC11996117

[55] P. Varela‐Centelles , J. Seoane , Y. Ulloa‐Morales , et al., “Oral Cancer Awareness in North‐Western Spain: A Population‐Based Study,” Medicina Oral Patología Oral y Cirugia Bucal 26, no. 4 (2021): e518–e525, 10.4317/medoral.24401. 34162825 PMC8254879

[56] C. Suárez‐Fernández , C. Barrientos , and M. García‐Pola , “Public Awareness on Oral Cancer: A Population‐Based Study in Asturias,” Asian Pacific Journal of Cancer Prevention 24, no. 12 (2023): 4127–4131, 10.31557/APJCP.2023.24.12.4127. 38156847 PMC10909085

[57] F. S. Jarab , W. Al‐Qerem , and R. Qarqaz , “Oral Cancer Awareness, Attitudes, and Barriers Among Jordanian Adults: A Cross‐Sectional Study,” Oral Health & Preventive Dentistry 20 (2022): 85–94, 10.3290/j.ohpd.b2805373. 35285596 PMC11641289

[58] C. Uguru , O. Chukwubuzor , U. Otakhoigbogie , U. Ogu , and N. Uguru , “Awareness and Knowledge of Risk Factors Associated With Oral Cancer Among Military Personnel in Nigeria,” Nigerian Journal of Clinical Practice 26, no. 1 (2023): 73–80, 10.4103/njcp.njcp_322_22. 36751827

[59] P. Rai , C. E. Goh , F. Seah , et al., “Oral Cancer Awareness of Tertiary Education Students and General Public in Singapore,” International Dental Journal 73, no. 5 (2023): 651–658, 10.1016/j.identj.2022.11.021. 36642572 PMC10509411

[60] I. Espinoza , Y. Serna , M. Fuentes , A. Jaramillo , K. Piedrahita , and G. Alvarez , “Oral Cancer Knowledge in Adults Evaluated Through a Phone Survey in the Context of the SARS‐CoV2 Health Emergency in Colombia,” Medicina Oral Patología Oral y Cirugia Bucal 28, no. 6 (2023): e630, 10.4317/medoral.26031. 37622430 PMC10635630

[61] Y. Fayaz , S. Ahmadi , A. Khawaja Omari , et al., “Oral Cancer Awareness Among Dental Students and Interns at Khatam Al Nabieen University, Kabul, Afghanistan,” Cancer Management and Research 16 (2024): 1727–1732, 10.2147/CMAR.S485942. 39664757 PMC11633300

[62] F. Kamal , E. Ghafary , M. H. Hamrah , et al., “Awareness and Knowledge of Tobacco Use and Its Relation to Oral Cancer Among Patients Visiting Stomatology Teaching Hospital,” Cancer Management and Research 16 (2024): 1345–1352, 10.2147/CMAR.S479933. 39380889 PMC11460352

[63] J. A. Chancellor , S. J. Ioannides , and J. M. Elwood , “Oral and Oropharyngeal Cancer and the Role of Sexual Behaviour: A Systematic Review,” Community Dentistry and Oral Epidemiology 45, no. 1 (2017): 20–34, 10.1111/cdoe.12255. 27642003

[64] C. Y. Chieng , A. Dalal , and V. Ilankovan , “Occupational Exposure and Risk of Oral and Oropharyngeal Squamous Cell Carcinoma: Systematic Review and 25‐year Retrospective Cohort Study of Patients,” British Journal of Oral and Maxillofacial Surgery 61, no. 1 (2023): 39–48, 10.1016/j.bjoms.2022.11.001. 36443129

[65] S. Lifsey , A. Cash , J. Anthony , S. Mathis , and S. Silva , “Building the Evidence Base for Population‐Level Interventions: Barriers and Opportunities,” supplement, Health Education & Behavior 42, no. 1 Suppl (2015): 133S–140SS, 10.1177/1090198114568429. 25829112

[66] C. F. Leask , M. Sandlund , D. A. Skelton , et al., “Framework, Principles and Recommendations for Utilising Participatory Methodologies in the Co‐Creation and Evaluation of Public Health Interventions,” Research Involvement and Engagement 5 (2019): 2, 10.1186/s40900-018-0136-9. 30652027 PMC6327557

[67] S. Tenny and M. R. Hoffman , Odds ratio. In: StatPearls [Internet] (Treasure Island (FL): StatPearls Publishing, 2023), https://www.ncbi.nlm.nih.gov/books/NBK431098/. 28613750

[68] Y. H. Lee , “An Overview of Meta‐Analysis for Clinicians,” Korean Journal of Internal Medicine 33, no. 2 (2018): 277–283, 10.3904/kjim.2016.195. 29277096 PMC5840596

[69] M. Alqahtani , A. Nahhas , L. Malibari , et al., “Awareness of Oral Cancer Among Dental Patients in Mecca,” Open Dentistry Journal 14, no. 1 (2020): 369–374.

[70] P. Pokhrel and B. Khadka , “Oral Cancer Awareness Among Undergraduate Dental Students of Kantipur Dental College and Hospital,” Journal of Nepal Health Research Council 18, no. 3 (2020): 541–543, 10.33314/jnhrc.v18i3.2873. 33210655

[71] R. Srivastava , S. Wazir , B. Jyoti , S. Kushwah , D. Pradhan , and P. Priyadarshi , “Perception and Outcome of Oral Cancer Awareness Among Clinical Undergraduate Dental Students of Tertiary Health Care Centre at Kanpur City: A Cross‐Sectional Study,” National Journal of Maxillofacial Surgery 11, no. 1 (2020): 89–93, 10.4103/njms.NJMS_6_19. 33041583 PMC7518484

[72] R. Nocini , G. Capocasale , D. Marchioni , and F. Zotti , “A Snapshot of Knowledge About Oral Cancer in Italy: A 505 Person Survey,” International Journal of Environmental Research and Public Health 17, no. 13 (2020): 4889, 10.3390/ijerph17134889. 32645880 PMC7370055

[73] S. Lakra , G. Kaur , A. Mehta , V. Kaushal , R. Atri , and Sunder , “Knowledge and Awareness of Oral Cancer Patients Regarding Its Etiology, Prevention, and Treatment,” Indian Journal of Dental Research 31, no. 4 (2020): 625–628, 10.4103/ijdr.IJDR_838_18. 33107467

[74] F. B. Lawal and O. F. Fagbule , “Knowledge of School‐Going Adolescents About the Oral Effects of Tobacco Usage in Ibadan, Southwest Nigeria,” International Quarterly of Community Health Education 40, no. 4 (2020): 337–343, 10.1177/0272684X19896730. 31865853

[75] P. Poudel , R. Srii , and V. Marla , “Oral Cancer Awareness Among Undergraduate Dental Students and Dental Surgeons: A Descriptive Cross‐Sectional Study,” Journal of Nepal Medical Association 58, no. 222 (2020): 102–107, 10.31729/jnma.4847. PMC7654458 32335622

[76] Y. S. Wimardhani , S. Warnakulasuriya , I. I. Wardhany , S. Syahzaman , Y. Agustina , and D. A. Maharani , “Knowledge and Practice Regarding Oral Cancer: A Study Among Dentists in Jakarta, Indonesia,” International Dental Journal 71, no. 4 (2021): 309–315, 10.1016/j.identj.2020.12.007. 33612266 PMC9275102

[77] B. Ojha , D. Bajracharya , and R. Baral , “Knowledge of Oral Cancer Among Online Respondent General Dentists: A Cross‐Sectional Survey,” Journal of Nepal Medical Association 59, no. 243 (2021): 1120–1124, 10.31729/jnma.5651. PMC9124318 35199772

[78] K. Schroeder , A. Panny , and N. Shimpi , “Community Awareness and Oral Cancer Screening in Rural Wisconsin,” Journal of Dental Hygiene: JDH 95, no. 4 (2021): 51–58. 34376544

[79] G. Keser , G. Yılmaz , and F. N. Pekiner , “Assessment of Knowledge Level and Awareness about Human Papillomavirus Among Dental Students,” Journal of Cancer Education 36, no. 4 (2021): 664–669, 10.1007/s13187-019-01683-3. 31898182

[80] O. Golburean , M. H. Hagen , D. Uncuta , et al., “Knowledge, Opinions, and Practices Related to Oral Cancer Prevention and Oral Mucosal Examination Among Dentists in Moldova, Belarus and Armenia: A Multi‐Country Cross‐Sectional Study,” BMC Oral Health 21, no. 1 (2021): 652, 10.1186/s12903-021-02011-2. 34922498 PMC8684171

[81] J. Oleszkiewicz‐Śpiołek , P. Adamska , G. Marvaso , B. A. Jereczek‐Fossa , P. Wychowanski , and A. Starzyńska , “Assessment of Awareness of Human Papillomavirus Infection Impact on Oral Cavity Among Patients,” Advances in Dermatology and Allergology 38, no. 6 (2021): 985–993, 10.5114/ada.2020.97396. 35126005 PMC8802954

[82] M. Tavakoli , M. Bater , and N. Taylor , “Current Knowledge and Awareness of Healthcare Professionals of Oral Cancer: A Study at a UK District General Hospital,” Journal of Cancer Education 36, no. 6 (2021): 1285–1289, 10.1007/s13187-020-01765-7. 32448924

[83] M. A. Alzabibi , H. Alolabi , D. A. Ali , et al., “Oral Cancer Knowledge and Practice Among Medical Students: A Cross‐Sectional Study During the Syrian Crisis,” Annals of Medicine & Surgery 77 (2022): 103504, 10.1016/j.amsu.2022.103504. 35638081 PMC9142404

[84] N. Saraswat , B. Everett , R. Pillay , N. Prabhu , A. Villarosa , and A. George , “Knowledge, Attitudes and Practices of Indian Immigrants in Australia Towards Oral Cancer and Their Perceived Role of General Practitioners: A Cross‐Sectional Study,” International Journal of Environmental Research and Public Health 19, no. 14 (2022): 8596, 10.3390/ijerph19148596. 35886448 PMC9319446

[85] K. Yadav , R. Hariprasad , R. Gupta , et al., “Cancer Awareness & Its Association With Demographic Variables & Mobile Phone Usage Among the Rural Population of a District in North India,” Indian Journal of Medical Research 156, no. 1 (2022): 94–103, 10.4103/ijmr.IJMR_3145_20. 36510902 PMC9903391

[86] L. Yang , A. Yang , L. N. Chen , N. Firth , S. R. Prabhu , and J. Zachar , “Knowledge of Oral Cancer Amongst Dental Patients Attending Public Clinics in South East Queensland, Australia,” Journal of Cancer Education 37, no. 4 (2022): 924–931, 10.1007/s13187-020-01901-3. 33068265

[87] R. M. Shadid , M. A. Abu Ali , and O. Kujan , “Knowledge, Attitudes, and Practices of Oral Cancer Prevention Among Dental Students and Interns: An Online Cross Sectional Questionnaire in Palestine,” BMC Oral Health 22, no. 1 (2022): 381, 10.1186/s12903-022-02415-8. 36064693 PMC9446528

[88] V. Vieira , G. Wendt , L. Ferreto , C. Pascotto , and L. Lucio , “University Students' Knowledge About the Relation Between Human Papillomavirus (HPV) and Head and Neck and Oral Cancers,” Asian Pacific Journal of Cancer Prevention 23, no. 8 (2022): 2719–2726, 10.31557/APJCP.2022.23.8.2719. 36037126 PMC9741904

[89] H. Y. Lee , Y. Luo , C. Daniel , K. Wang , and C. Ikenberg , “Is HPV Vaccine Awareness Associated With HPV Knowledge Level? Findings From HINTS Data Across Racial/Ethnic Groups in the US,” Ethnicity & Health 27, no. 5 (2022): 1166–1177, 10.1080/13557858.2020.1850648. 33307774

[90] S. Jo , K. A. Pituch , and N. Howe , “The Relationships Between Social Media and Human Papillomavirus Awareness and Knowledge: Cross‐Sectional Study,” JMIR Public Health and Surveillance 8, no. 9 (2022): e37274, 10.2196/37274. 36125858 PMC9533211

[91] P. Taneja , C. M. Marya , S. Jain , R. Nagpal , and S. Kataria , “Assessment of Knowledge, Attitude, and Practice Regarding Oral Cancer Among Dental Graduates—A Web‐Based Survey,” Journal of Cancer Education 37, no. 4 (2022): 1194–1200, 10.1007/s13187-020-01938-4. 33442863

[92] A. Murariu , E. R. Baciu , L. Bobu , et al., “Knowledge, Practice, and Awareness of Oral Cancer and HPV Infection Among Dental Students and Residents: A Cross‐Sectional Study,” Medicina 58, no. 6 (2022): 806, 10.3390/medicina58060806. 35744069 PMC9228335

[93] N. B. Fidele , S. M. N. Patrick , O. C. Okonji , and E. K. Kazadi , “Oral Cancer Awareness and Knowledge: Survey of Dentists in Democratic Republic of the Congo,” Journal of Cancer Policy 32 (2022): 100332, 10.1016/j.jcpo.2022.100332. 35560268

[94] D. Mavedatnia , K. Cuddy , H. Klieb , et al., “Oral Cancer Screening Knowledge and Practices Among Dental Professionals at the University of Toronto,” BMC Oral Health 23, no. 1 (2023): 343, 10.1186/s12903-023-03062-3. 37254183 PMC10230684

[95] S. Chatterjee , A. Mustafa Khan , R. Vj , et al., “Knowledge, Opinion, and Practices Towards Screening of Oral Cancer Among Homeopathy and Ayurveda Students in Indore, Madhya Pradesh, India,” Cureus 15, no. 3 (2023): e35707, 10.7759/cureus.35707. 37016645 PMC10066847

[96] R. M. Shadid and G. Habash , “Knowledge, Opinions, and Practices of Oral Cancer Prevention Among Palestinian Practicing Dentists: An Online Cross‐Sectional Questionnaire,” Healthcare 11, no. 7 (2023): 1005, 10.3390/healthcare11071005. 37046929 PMC10094010

[97] F. T. Alsulami , “Exploring the Impact of Knowledge About the Human Papillomavirus and Its Vaccine on Perceived Benefits and Barriers to Human Papillomavirus Vaccination Among Adults in the Western Region of Saudi Arabia,” Healthcare 12, no. 14 (2024): 1451, 10.3390/healthcare12141451. 39057593 PMC11276567

[98] R. Das , S. R. Misra , and A. Nayak , “Awareness Regarding Oral Cancer Amongst the Dental, Medical, and Nursing Students: Is Something Lacking,” Oral Oncology 152 (2024): 106790, 10.1016/j.oraloncology.2024.106790. 38569316

[99] K. K. Kanmodi , A. A. Salami , A. A. Gbadamosi , et al., “Strategies Adopted by Oral Physicians, Oral and Maxillofacial Surgeons, and Oral Pathologists in Patient Education on Oral Cancer: A Nigerian Study,” Cancer Reports 7, no. 1 (2024): e1929, 10.1002/cnr2.1929. 37884691 PMC10809197

[100] B. Lee and S. Mun , “Dental Hygienists' Knowledge, Performance Confidence and Awareness of Importance of Assessing Oral Cancer Risk Factors,” International Journal of Dental Hygiene 22, no. 4 (2024): 998–1007, 10.1111/idh.12815. 38659308

[101] A. G. M. Mahdi , “Knowledge and Attitude of Medical Students about Role of Human Papilloma Virus and Vaccine in Head and Neck Cancer,” Oral Oncology 156 (2024): 106939, 10.1016/j.oraloncology.2024.106939. 38991396

[102] M. A. Shubayr , M. M. A. Moaleem , S. A. Hakami , et al., “Factors Influencing Engagement in Oral Cancer Prevention Activities Among Dental Students and Professionals in Saudi Arabia,” BMC Oral Health 24, no. 1 (2024): 1465, 10.1186/s12903-024-05266-7. 39633404 PMC11619440

[103] R. Mroueh , T. Carpén , A. Mäkitie , et al., “Occupational Variation in the Incidence of Lip Cancer in the Nordic Countries,” Acta Oncologica 62, no. 6 (2023): 541–549, 10.1080/0284186X.2023.2224053. 37337140

[104] K. K. Kanmodi and P. A. Kanmodi , “A Call for the Inclusion of a Course on Basic Oral Healthcare Practice Into the Nigerian Nursing and Midwifery Education Curriculum,” Polish Annals of Medicine 28, no. 2 (2021): 256–258, 10.29089/2020.20.00162.

[105] I. M. Ponce‐Gonzalez , A. D. Cheadle , and M. L. Parchman , “Correlation of Oral Health Education by Community Health Workers With Changes in Oral Health Practices in Migrant Populations in Washington State,” Journal of Primary Care & Community Health 12 (2021): 21501327211002417, 10.1177/21501327211002417. PMC7968011 33719689

[106] P. Uwambaye and K. K. Kanmodi , “Incorporating Basic Periodontal Screening Into Antenatal Care Services Provided in Rwanda: A Policy Brief,” F1000Research 13 (2024): 647, 10.12688/f1000research.152760.2. 39193508 PMC11347909

[107] International Agency for Research on Cancer (IARC) Working Group on the Evaluation of Cancer‐Preventive Interventions . Oral Cancer Prevention (IARC, 2023), 19, https://publications.iarc.fr/Book-And-Report-Series/Iarc-Handbooks-Of-Cancer-Prevention/Oral-Cancer-Prevention-2023. 40273292

[108] A. Shamala , E. Halboub , S. A. Al‐Maweri , et al., “Oral Cancer Knowledge, Attitudes, and Practices Among Senior Dental Students in Yemen: A Multi‐Institution Study,” BMC Oral Health 23, no. 1 (2023): 435, 10.1186/s12903-023-03149-x. 37391820 PMC10314541

[109] B. C. Choi and A. W. Pak , “A Catalog of Biases in Questionnaires,” Preventing Chronic Disease 2, no. 1 (2005): 13. PMC1323316 15670466

[110] T. J. Wong , Q. Li , V. Dodd , W. Wang , J. Bian , and Y. Guo , “Oral Cancer Knowledge and Screening Behavior Among Smokers and Non‐Smokers in Rural Communities,” BMC Cancer 21, no. 1 (2021): 430, 10.1186/s12885-021-08198-5. 33879128 PMC8056680

[111] A. Dixit , N. H. Parekh , R. Anand , N. Kamal , A. Kumar , and B. K. Badiyani , “An Online Survey to Examine the Dental Students' Awareness, Knowledge, Prevention and Early Detection of Oral Cancer,” supplement, Journal of Pharmacy and BioAllied Sciences 15, no. Suppl 2 (2023): S984–S986, 10.4103/jpbs.jpbs_258_23. 37693960 PMC10485517

[112] M. M. Saab , S. FitzGerald , B. Noonan , et al., “Promoting Lung Cancer Awareness, Help‐Seeking and Early Detection: A Systematic Review of Interventions,” Health Promotion International 36, no. 6 (2021): 1656–1671, 10.1093/heapro/daab016. 33647930 PMC8699397

[113] J. Austoker , C. Bankhead , L. J. L. Forbes , et al., “Interventions to Promote Cancer Awareness and Early Presentation: Systematic Review,” British Journal of Cancer 101, no. Suppl 2 (2009): S31–S39, 10.1038/sj.bjc.6605388. 19956160 PMC2790702

[114] W. Olawole and K. Kanmodi , “Factors Responsible for Delayed Presentation at the Dental Clinic of the Federal Medical Centre, Birnin Kebbi, Nigeria,” Medical University 2, no. 1 (2019): 12–20.

[115] D. Winkeljohn , “Adherence to Oral Cancer Therapies: Nursing Interventions,” Clinical Journal of Oncology Nursing 14, no. 4 (2010): 461–466, 10.1188/10.CJON.461-466. 20682501

[116] K. Z. Siddall , S. N. Rogers , and C. J. Butterworth , “The Prosthodontic Pathway of the Oral Cancer Patient,” Dental Update 39, no. 2 (2012): 98–106, 10.12968/denu.2012.39.2.98. 22482267

[117] A. Natrah Aminnudin , J. G. Doss , S. Mazlipah Ismail , et al., “Can Post‐Treatment Oral Cancer Patients' Concerns Reflect Their Cancer Characteristics, HRQoL, Psychological Distress Level and Satisfaction With Consultation?,” Ecancermedicalscience 14 (2020): 1118, 10.3332/ecancer.2020.1118. 33209109 PMC7652548

[118] N. Shimpi , M. Jethwani , A. Bharatkumar , P. H. Chyou , I. Glurich , and A. Acharya , “Patient Awareness/Knowledge Towards Oral Cancer: A Cross‐Sectional Survey,” BMC Oral Health 18 (2018): 86, 10.1186/s12903-018-0539-x. 29764414 PMC5952627

[119] World Health Organization . WHO Global Strategy on People‐centred and Integrated Health Services: Interim Report. In WHO global strategy on people‐centred and integrated health services: interim report 2015, accessed, Jun 16, 2025, https://iris.who.int/bitstream/handle/10665/155002/WHO_HIS_SDS_2015.6_eng.pdf.

