# Supplementary material for: The Current State of Global Awareness and Knowledge on Oral Cancer: A Narrative Review
Source: Health Sci Rep. 2026 Feb 18;9(2):e71834. doi: 10.1002/hsr2.71834 (PMC12916887; doi:10.1002/hsr2.71834)
Supplement: Supplementary file 1 — Table S1: Summary of current primary studies reporting the sources of information on oral cancer among diverse population groups. Table S2: Summary of current primary studies reporting the prevalence and determinants of awareness and knowledge of the term “oral cancer” (or its subtype) among diverse population groups. Table S3: Summary of the included primary studies which investigated knowledge of the risk or aetiological factors of oral cancer among its study participants. Table S4: Summary of current primary studies reporting the prevalence and determinants of awareness and knowledge of the clinical features of oral cancer among diverse population groups. Table S5: Summary of the included primary studies which investigated the prevalence and determinants of knowledge of the preventability and preventative strategies of oral cancer among their study participants. Table S6: Summary of current primary studies reporting the prevalence and determinants of awareness and knowledge on oral cancer diagnostic approaches. Table S7: Summary of current primary studies reporting the prevalence and determinants of awareness and knowledge on the treatability and treatment options of oral cancer. [file HSR2-9-e71834-s001.docx]

**SUPPLEMENTARY FILE**

**Table S1. Summary of current primary studies reporting the sources of information on oral cancer among diverse population groups**

| **No.** | **Author(s) (Year)** | **Country (Location)** | **Setting** | **Study Population** | **Sample Size** | **Sample Characteristics** | **Specific Sources of Information on Oral Cancer** |
| --- | --- | --- | --- | --- | --- | --- | --- |
| 1 | do Prado et al. (2020) | Brazil (Recife) | Clinic | Patients | 251 | -64.5% were aged 18 to 50 years  -64.9% were females  -16.7% had college-level education  -11.6% were smokers  -42.6% were alcohol users | -Pharmacist (0.8%)  -Physician (7.5%)  -Dentist (18.3%)  -Public media [radio, newspaper, internet, magazine] (22.3%) |
| 2 | Yalcin & Gundogar (2020) | Turkey (Anatolia) | Clinic | Patients | 389 | -71.7% were aged ≤40 years  -53,7% were females  -41.4% had university-level education | -Medical doctor (12.1%)  -Newspaper (15.6%)  -Dentist (19.0%)  -Friends (31.1%)  -Television (50.1%)  -Internet (50.1%) |
| 3 | Zachar et al. (2020) | Australia (Bathurst, Orange, Dubbo, Albury-Wodonga, and Wagga  Wagga) | Clinic | Patients | 444 | -70.2% were aged >40 years  -60.9% were females  -61.0% were employed  -14.7% were current smokers  -77.1% were current alcohol users | -General medical practitioner (7.2%)  -Internet (14.0%)  -General dentist (20.7%)  -Relatives/friends/colleagues (21.2%)  -Magazine/Newspaper (15.2%)  -Cigarette package (35.7%)  -Radio/Television (33.1%) |
| 4 | Oswal et al. (2020) | India (Assam, Nagaland, Meghalaya) | Community | Community dwellers | 1400 | -71.1% were aged 44 years or below  -49% were females  -62% had secondary school-level or above education | -Colleges/Schools (17%)  -Social and public events (19%)  -Religious institutions (14%)  -Doctors/Nurses/Health workers (31%)  -Relatives/friends (47%)  -Media (71%) |
| 5 | Shahabudin et al. (2020) | Malaysia (Kedah) | Community | Community dwellers | 96 | -54.2% were aged ≥54.2 years  -95.8% had secondary school education  -88.5% were females  -68.8% were married | -Family (17.7%)  -Internet (27.1%)  -Health campaign (50.0%)  -Dental surgeon (51.0%)  -Media (54.2%) |
| 6 | Somathunga et al. (2021) | Sri Lanka (Peradeniya) | Clinic | Patients | 500 | -55% were females  -46.8% were aged between 14 and 34 years | -Family members (1.6%)  -Friends (1.6%)  -Midwife (1.8%)  -Newspaper (4.4%)  -Poster, banner (4.4%)  -Physician (4.8%)  -Dentist (5.8%)  -Radio or television (14.2%) |
| 7 | Adeoye et al. (2022) | China (Hong Kong) | Online | Community dwellers | 964 | -Aged between 18 and 86 years  -58.2% were females  -65.0% had tertiary level education  -6.4% were current tobacco users  -14.8% were former tobacco users  -15.7% were current alcohol drinkers  -7.0% were former alcohol drinkers | -Print materials (44.2%)  -Mass media outlets (48.6%)  -Internet platforms (53.8%) |
| 8 | Al Hulaibi et al. (2022) | Saudi Arabia (Jazan) | Online | Community dwellers | 400 | -45.8% were singles  -64.9% were females  -80% were aged 32 years or more  -52% were married  -56.4% dwelled in the village | -Mass media (58.4%)  -Family (20.6%)  -Dentist (12.3%)  -General physician (9.0%) |
| 9 | Ozdemir-Ozenen et al. (2022) | Turkey (Istanbul) | University | Students (dental) | 305 | -93.1% were aged 20 to 25 years  -26.5% were current smokers  -58.4% were females | -Academic curriculum (33.8%)  -Special courses (28.5%)  -Congresses (17.7%) |
| 10 | Tarakji (2022) | Saudi Arabia (multiple universities) | University | Students (dental) | 189 | -65% were males  -70% were aged 17 to 23 years  -72% were attending private universities | -Textbooks (63.0%)  -Academic curriculum (27%)  -Journals (6%)  -Dental conferences (4%) |
| 11 | Gerber et al. (2022) | Poland (Łódź; Wrocław), Germany (Dresden) | Clinic | Patients | 454 | -Aged 18 to 95 years  -64.5% were females  -85.2% had secondary or tertiary level of education  -49.1% were single  -31.9% resided in the village | -Friends (19.6%)  -Doctor (23.8%)  -Internet (33.7%)  -Television, radio, and newspapers (34.8%) |
| 12 | Zhou et al. (2022) | China (Beijing) | Community | Community dwellers | 3055 | -53.0% were aged 15 to 29 years  -54.2% were females  -53.7% were single  -81.5% had tertiary level education | -WeChat (social media) (13.0%)  -Neighbours and friends (15.5%)  -Phone news (20.3%)  -Television (25.2%) |
| 13 | Rupel et al. (2023) | Italy (Trieste) | University | Visitors | 750 | -54.41% were females  -Age ranged between 11 and 92 years  -64.75% were below 30 years of age | -Media (44.3%)  -Family and friends (34.5%)  -School (21.0%)  -Dentist (13.7%) |
| 14 | Deshpande et al. (2023) | India (Sawangi) | Clinic | Healthy relatives of cancer patients | 400 | -52.5% were aged below 35 years  -50.5% were females  -62.3% were married  -47.5% were university graduates | -Primary healthcare centre (1.3%)  -“Anganwadi” personnel (1.5%)  -Healthcare personnel (2.8%)  -Radio (3.8%)  -Magazines/books (5.3%)  -Newspaper (35.0%)  -Television (63.5%) |
| 15 | Dallak et al. (2024) | Saudi Arabia (Jazan) | Community | Community dwellers | 1380 | -52.9% were females  -63% were aged between 18 and 30 years  -45.7% were married  -50.6% lived in rural areas | -Dentist (13%)  -Physician (18%)  -Family and friends (23%)  -Media (41%) |
| 16 | Razavi et al. (2024) | Iran (Isfahan) | Clinic | Patients | 334 | -All participants were aged between 13 and 61 years  -59% were females  -61.1% were married  45.8% had formal education | -Social media (Instagram, Telegram, and WhatsApp) (26.3%)  -Acquaintances and friends (15.0%)  -Television and radio (12.6%)  -Medical personnel (10.2%) |

**Table S2. Summary of current primary studies reporting the prevalence and determinants of awareness and knowledge of the term “oral cancer” (or its subtype) among diverse population groups**

| **No.** | **Author(s) (Year)** | **Country (Location)** | **Setting** | **Study Population** | **Sample Size** | **Sample Characteristics** | **Prevalence of Awareness/ Knowledge of the term “Oral Cancer” (or its Subtype)** | **Determinants of Awareness/ Knowledge of the term “Oral Cancer” (or its Subtype)** |
| --- | --- | --- | --- | --- | --- | --- | --- | --- |
| 1 | Gunjal et al. (2020) | Malaysia (Petaling Jaya) | University | Students (medical and dental) | 557 | -63.0% were medical students  -37.0% were dental students  -54.7% were aged 22 to 24 years  -63.4% were females | -86.3% of the participating medical students were aware of the term “oral cancer”  -99.0% of the participating dental students were aware of the term “oral cancer”  -91.0% of the entire participants were aware of the term “oral cancer” | **Significant determinants:**  -Course of study (p<0.001)  **Non-significant determinants:**  -Age (p=0.655)  -Gender (p=0.881) |
| 2 | Bhat et al. (2020) | India (Bengaluru) | University | Students (non-health sciences) | 800 | -52% were males  -37.8% were in their third year of study | -41% to 55.5% were aware of the term “oral cancer” | No information on this was provided. |
| 3 | Oswal et al. (2020) | India (Assam, Nagaland, Meghalaya) | Community | Community dwellers | 1400 | -71.1% were aged 44 years or below  -49% were females  -62% had secondary school-level or above education | -59% were aware of the term “oral cancer” | No information on this was provided. |
| 4 | Kadashetti et al. (2020) | India (location not specified) | Clinic | Patients | 200 | -59% were males  -66% had secondary school education | -80% were aware of the term “oral cancer” | No information on this was provided. |
| 5 | Shahabudin et al. (2020) | Malaysia (Kedah) | Community | Community dwellers | 96 | -54.2% were aged ≥54.2 years  -95.8% had secondary school education  -88.5% were females  -68.8% were married | -77.1% were aware of the term “mouth cancer” | **Significant determinants:**  -Ethnicity (p=0.074)  -Occupation (p=0.0298)  **Non-significant determinants:**  -No information on this was provided. |
| 6 | Singh et al. (2020) | India (Bareilly) | Community | Community dwellers | 800 | -38.9% were aged between 18 and 27 years  -62.3% were males  -43.9% had formal education up to the tenth class | -71.80% were aware of the term “cancer of the mouth” | No information on this was provided. |
| 7 | Yalcin & Gundogar (2020) | Turkey (Anatolia) | Clinic | Patients | 389 | -71.7% were aged ≤40 years  -53,7% were females  -41.4% had university-level education | -23.1% were aware of the term “oral cancer” | No information on this was provided. |
| 8 | Dodd et al. (2021) | United Kingdom (London) | University | Students (specialty areas not specified) | 1415 | -Median age was 20 years  -55.9% were Caucasians  -87.6% were heterosexuals | -72.4% were aware of the term “oral cancer” | No information on this was provided. |
| 9 | Jafer et al. (2021) | Saudi Arabia (Jazan) | Clinic | Patients | 315 | -Mean age was 31 years  -58.8% were females  -45.7% had university level education | -57% were aware of oral cancer as a disease that affects the mouth  -81.7% knew that oral cancer is a malignant disease  -59% were knew that oral cancer is a disease that can metastasise to other body parts | No information on this was provided. |
| 10 | Muthanandam et al. (2021) | India (Lawspet) | Community | Community dwellers | 329 | -53.2% were females  -39.2% were aged between 31 and 60 years  -91% were not formally educated | -47.05% were aware of the term “oral cancer” | No information on this was provided. |
| 11 | Varela-Centelles et al. (2021) | Spain (Galicia) | Community | Community dwellers | 5727 | -30.2% were aged between 45 and 64 years  -47.7% were males | -When asked to name the cancer types they know, only: 3% of the participants named an oral cancer type in their first mention; only 8.2% named an oral cancer type in their first three mentions; and 20.3% mentioned an oral cancer type outside their first three mentions. | **Significant determinants (p-values were not stated in the article):**  -Gender  -Age  -Dental attendance (dental clinic use)  -Educational achievements  -History of smoking  -History of alcohol use |
| 12 | Al Hulaibi et al. (2022) | Saudi Arabia (Jazan) | Online | Community dwellers | 400 | -45.8% were singles  -64.9% were females  -80% were aged 32 years or more  -52% were married  -56.4% dwelled in the village | -87.3% were aware of the term “oral cancer” | No information on this was provided. |
| 13 | Jarab et al. (2022) | Jordan (location not specified) | Online | Community dwellers | 1307 | -Mean age was 26.10 years  -77.35% were females  -77.9% were not married  -90.1% had bachelor’s degree education or higher | -70% were aware of the term “oral cancer” | **Significant determinants:**  -Professional field (p<0.001)  -Source of information (p=0.004)  -Age (0.010)  **Non-significant determinants:**  -Marital status (p=0.775)  -Smoking status (p=0.349)  -Educational level (p=0.263)  -Sex (p=0.419)  -Area of residence (p=0.096)  -Average household monthly income (p=0.163)  -Acquaintance with someone with cancer (p=0.540) |
| 14 | Zhou et al. (2022) | China (Beijing) | Community | Community dwellers | 3055 | -53.0% were aged 15 to 29 years  -54.2% were females  -53.7% were single  -81.5% had tertiary level education | -52.9% were aware of the term “oral cancer” | **Significant determinants:**  -Place of residence (p=0.015)  -History of betel nut chewing (p=0.010)  -Mouth self-examination practices  **Non-significant determinants:**  -No information on this was provided. |
| 15 | Suárez-Fernández et al. (2023) | Spain (Asturias) | Online | Community dwellers | 676 | -71.2% were females  -75.9% were aged between 18 and 50 years  -75.9% were not health professionals | -When asked to name the cancer types they know, only: 4.3% of the participants named an oral cancer type in their first mention; only 9.6% named an oral cancer type in their first three mentions; and 24.8% mentioned an oral cancer type outside their first three mentions. | No information on this was provided. |
| 16 | Anirudh et al. (2023) | India (specific location not mentioned) | Clinic | Patients | 158 | -61% were females  -39.2% were aged between 46 and 60 years  -46% had secondary school education | -67.1% were aware of the term “oral cancer” | No information on this was provided. |
| 17 | Rupel et al. (2023) | Italy (Trieste) | University | Visitors | 750 | -54.41% were females  -Age ranged between 11 and 92 years  -64.75% were below 30 years of age | -68.4% were aware of the term “oral cancer” | **Significant determinants:**  -Level of education (p=0.01)  -Gender (p=0.02)  **Non-significant determinants:**  -No information on this was provided. |
| 18 | Uguru et al. (2023) | Nigeria (Enugu) | Military | Army personnel | 300 | -All were aged between 19 and 59 years  -Mean age was 37.5 years  -79.7% were males  -41.7% had tertiary level of education  -61.7% had 1 to 15 years’ experience in the army | -15.35% were aware of the term “oral cancer” | No information on this was provided. |
| 19 | Chan et al. (2023) | Malaysia (13 dental schools in Malaysia) | Online | Students (dental) | 595 | -56% were females  -77.1% wee aged between 22 and 24 years | -99.5% were aware of the term “oral cancer” | **Significant determinants:**  -Level of academic year (p<0.001)  -School type (private versus public) (p=0.002)  -Gender (p=0.037)  **Non-significant determinants:**  -Age (p=0.145). |
| 20 | Rai et al. (2023) | Singapore (location not specified) | University and community | Students (dental, medical, nonmedical, and nondental) and nonstudents (community dwellers) | 470 | -62% were females  -83% were Chinese  -20% were dental students  -13% were medical students  -35% were nonmedical and nondental students  -32% were members of the general public | -95% of medical and dental students were aware of the term “oral cancer”  -66% of the general public, and nonmedical and nondental students were aware of the term “oral cancer” | No information on this was provided. |
| 21 | Chugh et al. (2023) | India (Rajasthan) | Clinic | Patients | 446 | -83.6% were males  -63.1% were in the low socioeconomic status  -40.8% belonged to the age group of 21 to 30 years | -20% were aware of the term “oral cancer” | No information on this was provided. |
| 22 | Alsalhani et al. (2024) | Saudi Arabia  (location not specified) | University | Students (dental) and Dentists (interns) | 451 | -64.7% were aged between 21 and 25 years  -58.5% were males  -52.8% were dental students | -90.2% were aware of the term “oral cancer” | No information on this was provided. |
| 23 | Dallak et al. (2024) | Saudi Arabia (Jazan) | Community | Community dwellers | 1380 | -52.9% were females  -63% were aged between 18 and 30 years  -45.7% were married  -50.6% lived in rural areas | -74.1% were aware of the term “oral cancer” | No information on this was provided. |
| 24 | Espinoza et al. (2024) | Colombia (Antioquia) | Clinic | Patients | 268 | -71.3% were females  -33.6% were from low socioeconomic stratum  -61.2% had up to middle school education | -58.6% were aware of the term “oral cancer” | No information on this was provided. |
| 25 | Fayaz et al. (2024) | Afghanistan (Kabul) | University | Students (dental) and dentists (interns) | 453 | -60.7% were females  -42.6% were fifth-year dental students | -88.07% were aware of the term “oral cancer” | No information on this was provided. |
| 26 | Kamal et al. (2024) | Afghanistan (Kabul) | Clinic | Patients | 435 | -All were aged between 15 and 76 years  -79.1% were males | -41.8% were aware of the term “oral cancer” | No information on this was provided. |

**Table S3. Summary of the included primary studies which investigated knowledge of the risk or aetiological factors of oral cancer among its study participants**

| **No.** | **Author(s) (Year)** | **Country (Location)** | **Setting** | **Study Population** | **Sample Size** | **Sample Characteristics** | **Rate of Awareness/Knowledge of the Aetiological/Risk Factors of Oral Cancer** | **Determinant of Awareness/Knowledge of the Aetiological/Risk of Oral Cancer** |
| --- | --- | --- | --- | --- | --- | --- | --- | --- |
| 1 | Alqahtani et al. (2020) | Saudi Arabia (Mecca) | Clinic | Patients | 416 | -71.4% were females  -57.7% were aged between 18 and 30 years  -44.5% had educational level at high school level or lower | -Exposure to the sun (3.9%)  -Eating of spicy food (4.9%)  -Human papillomavirus infection (6.9%)  -Poor diet (7.8%)  -Poor oral hygiene (11.8%)  -Alcohol use (13.7%)  -Use of smokeless tobacco (18.6%)  -Smoking (19.6%)  -Sore denture (45.1%)  -Improper flossing and brushing technique (75.5%) | No information on this was provided. |
| 2 | Pokhrel & Khadka (2020) | Nepal (Kathmandu) | University | Students (Dental) | 101 | -20.8% were males  -Mean age of all were 22.28 years  -48.5% were either in their third or fourth year | -Alcohol use (20.8%)  -Human papillomavirus infection (40.6%)  -Smokeless tobacco use (56.4%)  -Tobacco smoking (86.1%) | No information on this was provided. |
| 3 | Srivastava et al. (2020) | India (Kanpur) | University | Students (Dental) | 139 | -56.1% were interns  -56.8% were females | -Alcohol with tobacco (0.7%)  -Pan masala (4.3%)  -Pan masala with tobacco (4.3%)  -Sharp teeth (14.4%)  -Tobacco (75.5%)  -Alcohol (75.5%) | No information on this was provided. |
| 4 | Singh et al. (2020) | India (Bareilly) | Community | Community dwellers | 800 | -38.9% were aged between 18 and 27 years  -62.3% were males  -43.9% had formal education up to the tenth class | -Erythroplakia and/or leukoplakia (43.3%) | No information on this was provided. |
| 5 | Nocini et al. (2020) | Italy (location not specified) | Online | Community dwellers and patients | 505 | -71.49% were females  -All were aged between 18 and 76 years  -44.75% had high school diploma | -Human papillomavirus infection (54.65%)  -Alcohol use (58.01%%)  -Poor oral hygiene (60.59%)  -Smoking (89.30%) | No information on this was provided. |
| 6 | Oswal et al. (2020) | India (Assam, Nagaland, Meghalaya) | Community | Community dwellers | 1400 | -71.1% were aged 44 years or below  -49% were females  -62% had secondary school-level or above education | -Alcohol use (41%)  -Use of supari/areca/betel nut (66%)  -Tobacco use (75%) | No information on this was provided. |
| 7 | Bhat et al. (2020) | India (Bengaluru) | University | Students (non-health sciences) | 800 | -52% were males  -37.8% were in their third year of study | -Human papillomavirus infection (<12%)  -Ultraviolet radiation (<25%)  -Chronic oral trauma from tooth (<25%)  -Hot and spicy food (<25%)  -Alcohol use (25%)  -Smokeless tobacco (>50%)  -Tobacco smoking (>60%) | No information on this was provided. |
| 8 | Kadashetti et al. (2020) | India (location not specified) | Clinic | Patients | 200 | -59% were males  -66% had secondary school education | -Nutritional deficiency (12%)  -Alcohol use (14%)  -Smoking (29%)  -Betel quid chewing (58%) | No information on this was provided. |
| 9 | Lakra et al. (2020) | India (Rohtak) | Clinic | Patients | 218 | -88.1% were males  -53.3% were in upper lower class  -Mean age of all participants was 55.7 years | -Alcohol and/or tobacco (55.5%) | No information on this was provided. |
| 10 | Shahabudin et al. (2020) | Malaysia (Kedah) | Community | Community dwellers | 96 | -54.2% were aged ≥54.2 years  -95.8% had secondary school education  -88.5% were females  -68.8% were married | -Excessive sugar intake (15.6%)  -Excessive exposure to ultraviolet radiation (19.8%)  -Eating of spicy food (27.1%)  -Chewing of betel quid (35.4%)  -Alcohol use (38.5%)  -Smoking (64.6%) | No information on this was provided. |
| 11 | Zachar et al. (2020) | Australia (Bathurst, Orange, Dubbo, Albury-Wodonga, and Wagga  Wagga) | Clinic | Patients | 444 | -70.2% were aged >40 years  -60.9% were females  -61.0% were employed  -14.7% were current smokers  -77.1% were current alcohol users | -Human papillomavirus infection (24.0%)  -Older age (35.8%)  -Stress (38.5%)  -Poor diet (52.6%)  -Alcohol use (57.1%)  -Family history (63.5%)  -Poor oral hygiene (77.2%)  -Smoking (96.4%) | **Significant determinants:**  -Family history (p=0.047)  -Age (p=0.002)  **Non-significant determinants:**  No information on this was provided. |
| 12 | do Prado et al. (2020) | Brazil (Recife) | Clinic | Patients | 251 | -64.5% were aged 18 to 50 years  -64.9% were females  -16.7% had college-level education  -11.6% were smokers  -42.6% were alcohol users | -Consumption of spicy foods (3.6%)  -Obesity (4.8%)  -Use of cell phone (5.6%)  -Ultraviolet radiation (12.0%)  -Illicit drug use (12.8%)  -Removable dentures (15.1%)  -Alcohol use (25.1%)  -Infectious diseases (38.3%)  -Smoking (48.6%) | No information on this was provided. |
| 13 | Gunjal et al. (2020) | Malaysia (Petaling Jaya) | University | Students (medical and dental) | 557 | -63.0% were medical students  -37.0% were dental students  -54.7% were aged 22 to 24 years  -63.4% were females | *D denotes dental students while M denotes medical students.  -Smoking (88.3% [M], 99% [D])  -Smokeless tobacco (64.1% [M], 92.7% [D])  -Alcohol (61.5% [M], 91.7% [D])  -Betel quid chewing (57.7% [M], 99% [D])  -Family history (72.9% [M], 95.1% [D])  -Excessive sunlight (21.1% [M], 83.5% [D])  -Spicy foods (47.1 [M], 56.3% [D])  -Red or processed meat (43.4% [M], 40.3% [D])  -Human papillomavirus infection (53.7% [M], 90.3% [D])  -Inadequate fruits and vegetable consumption (33.7% [M], 45.6% [D])  -Chronic irritation (59.1% [M], 79.1% [D])  -Radiation (84% [M], 96.6% [D]), Immunosuppression (76.9% [M], 93.7% [D]) | No information on this was provided. |
| 14 | Lawal & Fagbule (2020) | Nigeria (Ibadan) | Secondary school | Students (senior) | 1465 | -All were aged between 12 and 20 years  -50.3% were males | -Tobacco use (1.1%) | No information on this was provided. |
| 15 | Poudel et al. (2020) | Nepal (Kathmandu) | University | Students (dental) and dentists | 508 | -24.4% were in their third year of study  -3.1% were dentists | -Systemic diseases (0.39%)  -Immunosuppression (2.1%)  -Dietary factors (3.74%)  -Ultraviolet radiation (8.66%)  -Poor oral hygiene (10.43%)  -Genetic factors (12.20%)  -Viral infection (14.17%)  -Alcohol use (20.86%)  -Tobacco chewing (77.75%)  -Smoking (89.96%) | No information on this was provided. |
| 16 | Yalcin & Gundogar (2020) | Turkey (Anatolia) | Clinic | Patients | 389 | -71.7% were aged ≤40 years  -53,7% were females  -41.4% had university-level education | -Heavy exposure to the sun (12.1%)  -Eating of spicy food (16.9%)  -Lip or cheek biting (21.2%)  -Older age (21.2%)  -Drinking or eating hot food items (38.9%)  -Tobacco use (67.9%)  -Alcohol use (68.7%)  -Smoking (95.5%) | No information on this was provided. |
| 17 | Jafer et al. (2021) | Saudi Arabia (Jazan) | Clinic | Patients | 315 | -Mean age was 31 years  -58.8% were females  -45.7% had university level education | -Genetic factors (79.2%)  -Smoking (82%) | No information on this was provided. |
| 18 | Wimardhani et al. (2021) | Indonesia (Jakarta) | Clinic | Dentists | 402 | -79.6% were females  -37.8% were aged between 30 and 49 years  -22.9% were from the eastern region of Jakarta | -Lip cancer related to sun exposure (34.8%)  -Use of smokeless tobacco (54.2%)  -Older age (55.2%)  -Inadequate consumption of vegetables and fruits (70.1%)  -Human papillomavirus infection (79.6%)  -Prior history of oral cancer (91.0%)  -Alcohol use (92.0%)  -Tobacco use (95.5%) | No information on this was provided. |
| 19 | Ojha et al. (2021) | Nepal (Kathmandu) | Clinic | Dentists | 234 | -68.5% were females  -Mean age was 27 years | -Alcohol (41.5%)  -Family history of oral cancer (46.2%)  -Immunosuppression (53.8%)  -Smoked tobacco use (76.1%)  -Smokeless tobacco use (78.6%) | No information on this was provided. |
| 20 | Schroeder et al., 2021 | United States of America (Wisconsin) | Agricultural setting (farms) | Farmers | 236 | -55% were females  -78% were aged between 51 and 80 years  -23.0% had university bachelor’s degree education or above | -Prolonged exposure to the sun (28.0%)  -Human papillomavirus infection (28.0%)  -Smoking and alcohol use (38.0%)  -Alcohol use (41.0%)  -Tobacco use (71.0%) | No information on this was provided. |
| 21 | Muthanandam et al. (2021) | India (Lawspet) | Community | Community dwellers | 329 | -53.2% were females  -39.2% were aged between 31 and 60 years  -91% were not formally educated | -Alcohol use (29.15%)  -Tobacco chewing (62.5%)  -Smoking (68.05%) | No information on this was provided. |
| 22 | Dodd et al. (2021) | United Kingdom (London) | University | Students (specialty areas not specified) | 1415 | -Median age was 20 years  -55.9% were Caucasians  -87.6% were heterosexuals | Human papillomavirus infection (26.7%) | **Significant determinants:**  -Gender (p<0.05)  -Alcohol use (p<0.05)  -Sexuality (heterosexual/non-heterosexual) (p<0.05)  -Sexual activity (p<0.05)  -Intent to receive vaccination against human papillomavirus infection in the future (p<0.05)  **Non-significant determinants:**  -History of sexually transmitted infections (p>0.05) |
| 23 | Keser et al. (2021) | Turkey (location was not specified) | University | Students (dental) | 318 | -68.9% were females  -Mean age was 22.87 years  -31.4% were in their third year of study | -Human papillomavirus infection (82.7%) | No information on this was provided. |
| 24 | Golburean et al. (2021) | Moldova (Chisinau), Belarus (Minsk), Armenia, and (Yerevan) | Clinic | Dentists | 1316 | -66% were males  -32.7% had between 5 and 15 years of experience in dental practice  -41.5% were general dentists | -Inadequate consumption of vegetables and fruits (>28%)  -Older age (>44%)  -Alcohol abuse (>49%)  -Human papillomavirus infection (>60.9%)  -Prior history of oral cancer (>83%)  -Tobacco use (>82%) | No information on this was provided. |
| 25 | Oleszkiewicz-Śpiołek et al. (2021) | Poland (Gdańsk) | Clinic | Patients | 663 | -Mean age was 21 years  -All were aged between 18 and 65 years  -58.13% were females | -Human papillomavirus infection (45.45%) | No information on this was provided. |
| 26 | Tavakoli et al. (2021) | United Kingdom (location not specified) | Clinic | Clinical staff (medical doctors, nurses, and healthcare assistants) | 155 | -11% were medical doctors  -61.3% were nurses  -27.7% were healthcare assistants | -Vaping (47%)  -Dental caries (51%)  -Human papillomavirus (59%)  -Alcohol use (64%)  -Tobacco smoking (98%) | No information on this was provided. |
| 27 | Alzabibi et al. (2022) | Syria (Damascus) | University | Students (medical) | 301 | -All were aged between 18 and 30 years  -61.15% were males  -65.5% were in the clinical phase of their academic programme | -Low fibre diet (15.6%)  -Hot and spicy food (41.2%)  -Sunlight (49.5%)  -Old age (51.8%)  -Alcohol use (68.4%)  -Viral infection (69.4%)  -Smoking (95.3%) | No information on this was provided. |
| 28 | Al Hulaibi et al. (2022) | Saudi Arabia (Jazan) | Online | Community dwellers | 400 | -45.8% were singles  -64.9% were females  -80% were aged 32 years or more  -52% were married  -56.4% dwelled in the village | -Exposure to sunlight (14%)  -Family history of oral cancer (47.3%)  -Alcohol use (74.4%)  -Sniffing (84.8%)  -Smoking (89.7%) | No information on this was provided. |
| 29 | Jarab et al. (2022) | Jordan (location not specified) | Online | Community dwellers | 1307 | -Mean age was 26.10 years  -77.35% were females  -77.9% were not married  -90.1% had bachelor’s degree education or higher | -Prolonged exposure to the sun (20.66%)  -Biological sex (36.5%)  -Older age (47.98%)  -Poor diet (56.17%)  -Human papillomavirus infection (64.48%)  -Poor oral hygiene (67.98%)  -Alcohol intake (83.28%)  -Smoking (92.79%) | No information on this was provided. |
| 30 | Saraswat et al. (2022) | Australia (eight states in Australia) | Online and phone | Community dwellers | 164 | -Mean age was 35.2 years  -52.8% were males  -47.5% were resident of New South Wales  -90% had university education | -Family history (41.8%)  -Heavy alcohol drinking (64.7%)  -Betel quid chewing (75.8%)  -Betel nut chewing (77.8%)  -Shisha smoking (86.9%)  -Tobacco, cigar, or pipe smoking (92.2%)  -Tobacco chewing (94.1%) | No information on this was provided. |
| 31 | Yadav et al. (2022) | India (Gautam Buddh Nagar) | Community | Community dwellers | 204 | -70.6% were females  -56.4% were aged between 18 and 30 years  -35.3% were formally educated up to middle school  -51.9% were unemployed | -Excessive chewing of gum (0.5%)  -Poor oral hygiene (8.3%)  -Tobacco use (71.6%) | No information on this was provided. |
| 32 | Yang et al. (2022) | Australia (Queensland) | Clinic | Patients | 213 | -51.3% were aged above 60 years  -54.4% were females  -53.8% were educated to tertiary level | -Ill-fitted dentures (≈12.5%)  -Vitamin deficiencies (≈31%)  -Old age (≈40%)  -Alcohol use (53.8%)  -Family history of oral cancer (57.5%)  -Smoking (84.4%) | **Significant determinants:**  -Level of education (p=0.001)  -Family history of oral cancer (p=0.004)  -Gender (0.008)  **Non-significant determinants:**  No information on this was provided. |
| 33 | Gerber et al. (2022) | Poland (Łódź; Wrocław), Germany (Dresden) | Clinic | Patients | 454 | -Aged 18 to 95 years  -64.5% were females  -85.2% had secondary or tertiary level of education  -49.1% were single  -31.9% resided in the village | -Nutrition and diet (22.7%)  -Overexposure to sunlight (41.9%)  -Human papillomavirus infection (39.2%)  -Age (35.5%)  -Alcohol use (69.8%)  -Tobacco use (84.4%) | No information on this was provided. |
| 34 | Shadid et al. (2022) | Palestine (West Bank) | University | Dental interns and students (dental) | 392 | -94.1% were aged 30 years or below  -64.3% were females  -10.5% were interns | -Family history of oral cancer (7.4%)  -Low intake of vegetables and fruits (26.5%)  -Poor oral hygiene (26.5%)  -Intake of spicy foods (47.7%)  -Consumption of hot foods and beverages (49.2%)  -Human papillomavirus infection (70.2%)  -Exposure to sunlight (76.5%)  -Alcohol use (80.6%)  -Older age (81.6%)  -Prior history of oral cancer (84.7%)  -Use of tobacco products (88.8%) | No information on this was provided. |
| 35 | Vieira et al. (2022) | Brazil (Paraná) | University | Students (non-dental) | 335 | -29% were medical (human medicine) students while others (71%) were non-health science students  -All were aged between 16 and 55 years  -74% were women | -Human papillomavirus infection (65.1%) | No information on this was provided. |
| 36 | Zhou et al. (2022) | China (Beijing) | Community | Community dwellers | 3055 | -53.0% were aged 15 to 29 years  -54.2% were females  -53.7% were single  -81.5% had tertiary level education | -Drinking (25.5%)  -Age (28.0%)  -Smoking (39.6%)  -Betel nut chewing (41.9%) | No information on this was provided. |
| 37 | Lee et al. (2022) | United States of America (multiple locations; locations not specified) | Community | Community dwellers | 3533 | -51.615 were aged 46 years or above  -59.16% were females  -60.38% did not attain bachelor’s degree or above level of education  -34.24% were unemployed | -Human papillomavirus infection (28.78%) | No information on this was provided. |
| 38 | Jo et al. (2022) | United States of America (multiple locations; locations not specified) | Community | Community dwellers | 2948 | -57.36% were females  -57.53% had post-secondary school education  -45.22% were employed  -17.91% were single | -Human papillomavirus infection (19.20%) | No information on this was provided. |
| 39 | Taneja et al. (2022) | India (location not specified) | University | Dentists | 205 | -Mean age was 23.02 years  -57.2% were females | -Consumption of hot foods and beverages (54%)  -Low intake of fruits (56%)  -Ill-fitting dentures (62%)  -Consumption of spicy foods (64%)  -Poor oral hygiene (64%)  -Family history of cancer (68%)  -Alcohol use (69%)  -Older age (70%)  -Use of tobacco products (71%) | No information on this was provided. |
| 40 | Murariu et al. (2022) | Romania (Iasi) | University | Dentists and students (dental) | 197 | -Mean age was 25.01 years  -64.0% were females  -28.9% were resident dentists | -Old age (>6%)  -Dietary factors (>6%)  -Chronic irritation of the oral mucosa (>15%)  -Ultraviolet radiation exposure (>25%)  -Human papillomavirus infection (>28%)  -Alcohol use (>56%)  -Tobacco use (>87%) | No information on this was provided. |
| 41 | Fidele et al. (2022) | Democratic Republic of Congo (locations not specified) | Clinic | Dentists | 162 | -54.3% were aged between 30 and 39 years  -65.4% were males  -81.5% were in general dental practice | -Oral sex (≈30%)  -Intake of spicy foods (≈30%)  -Obesity (≈30%)  -Exposure to sunlight (≈36%)  -Human papillomavirus infection (≈45%)  -Autoimmune conditions (≈50%)  -Ill-fitting denture use (≈62%)  -Older age (≈60%)  -Family history of oral cancer (≈70%)  -Prior history of cancer (≈80%)  -Use of tobacco products (≈80%)  -Poor oral hygiene (≈90%)  -Alcohol use (≈100%) | No information on this was provided. |
| 42 | Tarakji (2022) | Saudi Arabia (multiple universities) | University | Students (dental) | 189 | -65% were males  -70% were aged 17 to 23 years  -72% were attending private universities | -Inadequate intake of vegetables and fruits (53%)  -Old age (65.6%)  -Ultraviolet radiation exposure (72.5%)  -Human papillomavirus infection (77.8%)  -Alcohol use (83.6%)  -Having premalignant lesions (85.2%)  -Tobacco use (87%) | No information on this was provided. |
| 43 | Ozdemir-Ozenen et al. (2022) | Turkey (Istanbul) | University | Students (dental) | 305 | -93.1% were aged 20 to 25 years  -26.5% were current smokers  -58.4% were females | -Being of a male sex (30.2%)  -Inadequate intake of vegetables and fruits (52.8%)  -Older age (60.3%)  -Intake of hot and spicy foods (61.3%)  -Alcohol use (75.1%)  -Exposure to sunlight (76.1%)  -Frequent biting of the lips and cheeks (80.7%)  -Tobacco use (82%)  -Poor oral hygiene (85.6%)  -Family history of head and neck cancer (86.6%)  -Prior history of oral cancer (87.9%) | No information on this was provided. |
| 44 | Mavedatnia et al. (2023) | Canada (Toronto) | University | Dentists | 91 | -61.5% were males  -87.9% were practicing in large urban areas | -Alcohol use (65.6%)  -Betel nut use (75.6%)  -Smoking (98.9%) | No information on this was provided. |
| 45 | Uguru et al. (2023) | Nigeria (Enugu) | Military | Army personnel | 300 | -All were aged between 19 and 59 years  -Mean age was 37.5 years  -79.7% were males  -41.7% had tertiary level of education  -61.7% had 1 to 15 years’ experience in the army | -Bad food (8.0%)  -Sex (14.0%)  -Enemy (15.0%)  -Smoking (16.0%)  -Spiritual attack (18.0%)  -Kissing (29.0%) | No information on this was provided. |
| 46 | Rai et al. (2023) | Singapore (location not specified) | University and community | Students (dental, medical, nonmedical, and nondental) and nonstudents (community dwellers) | 470 | -62% were females  -83% were Chinese  -20% were dental students  -13% were medical students  -35% were nonmedical and nondental students  -32% were members of the general public | -Viral infections (46%)  -Betel quid chewing (47%)  -Alcohol use (49%)  -Smoking (92%) | No information on this was provided. |
| 47 | Rupel et al. (2023) | Italy (Trieste) | University | Visitors | 750 | -54.41% were females  -Age ranged between 11 and 92 years  -64.75% were below 30 years of age | -Fluoride use (12.7%)  -Exposure to sunlight (15.4%)  -Amalgam fillings (34.7%)  -Alcohol use (51.4%)  -Smoking (94.1%) | No information on this was provided. |
| 48 | Deshpande et al. (2023) | India (Sawangi) | Clinic | Healthy relatives of cancer patients | 400 | -52.5% were aged below 35 years  -50.5% were females  -62.3% were married  -47.5% were university graduates | -Betel nut use (≈2%)  -Bidi use (≈5%)  -Gutka use (≈6.25%)  -Tobacco use (≈42.5%)  -Cigarette use (≈45%) | No information on this was provided. |
| 49 | Anirudh et al. (2023) | India (specific location not mentioned) | Clinic | Patients | 158 | -61% were females  -39.2% were aged between 46 and 60 years  -46% had secondary school education | -Inadequate intake of vegetables and fruits (1.9%)  -Infection (1.9%)  -Hereditary factors (2.5%)  -Old age (2.5%)  -Poor oral health (3.2%)  -Smoking (12.7%)  -Pan chewing (31%)  -Alcohol use (44.3%) | No information on this was provided. |
| 50 | Chugh et al. (2023) | India (Rajasthan) | Clinic | Patients | 446 | -83.6% were males  -63.1% were in the low socioeconomic status  -40.8% belonged to the age group of 21 to 30 years | -Genetic factors (≈1.1%)  -Eating of spicy foods (≈1.1%)  -Inadequate consumption of green vegetables (≈9.0%)  -Chronic irritation from denture or sharp tooth (≈10.0%)  -Poor oral hygiene (≈12.3%)  -Alcohol use (≈13.0%)  -Tobacco use (≈15.7%) | No information on this was provided. |
| 51 | Chatterjee et al. (2023) | India (Indore) | University | Students (homeopathy and ayurveda) | 310 | -Mean age was 23.84 years  -52.3% were females  -32.9% were in their third year of academic programme | -Old age (12.9%)  -Alcohol use (60%)  -Tobacco use (99.7%) | No information on this was provided. |
| 52 | Shadid & Habash (2023) | Palestine (locations not specified) | Clinical | Dentists | 254 | -65.7% were aged above 30 years  -43.7% were females  -42.5% had 6 to 15 years of experience  -80.7% worked in a private setting  -79.9% were general dentists | -Family history of oral cancer (10.2%)  -Obesity (19.3%)  -Ill-fitted denture use (25.6%)  -Poor oral hygiene (31.1%)  -Consumption of spicy foods (49.6%)  -Consumption of hot foods and beverages (65.7%)  -Older age (67.3%)  -Human papillomavirus infection (74.8%)  -Exposure to sun (86.2%)  -Prior history of oral cancer (92.9%)  -Alcohol use (92.9%)  -Use of tobacco products (97.2%) | No information on this was provided. |
| 53 | Alsalhani et al. (2024) | Saudi Arabia  (location not specified) | University | Students (dental) and Dentists (interns) | 451 | -64.7% were aged between 21 and 25 years  -58.5% were males  -52.8% were dental students | -Human papillomavirus infection (62.3%) | No information on this was provided. |
| 54 | Alsulami (2024) | Saudi Arabia  (Mecca) | Online | Community dwellers | 1149 | -Mean age was 34.35 years  -80.9% were females  -58.4% were married  -60% were unemployed | -Human papillomavirus infection (20.5%) | No information on this was provided. |
| 55 | Dallak et al. (2024) | Saudi Arabia (Jazan) | Community | Community dwellers | 1380 | -52.9% were females  -63% were aged between 18 and 30 years  -45.7% were married  -50.6% lived in rural areas | The information below was concerning those participants (n=1022) who were aware of oral cancer:  -Obesity (11.9%)  -Exposure to the sun (12.9%)  -Eating of spicy and hot foods (13.8%)  -Ill-fitting denture (14.7%)  -Eating of canned food (16%)  -Vitamin deficiency (19%)  -Old age (32.3%)  -Human papillomavirus infection (33.5%)  -Family history of oral cancer (47.6%)  -Poor oral hygiene (48.3%)  -Alcohol use (58.9%)  -Khat use (62.1%)  -Use of smokeless tobacco (87%)  -Smoking (86.4%) | No information on this was provided. |
| 56 | Das et al. (2024) | India (Odisha) | University | Students (nursing, medical, and dental) | 1000 | -23%, 35.4%, and 41.6% were nursing, medical, and dental students respectively | -Alcohol and tobacco use (93%)  -Human papillomavirus infection (72.7%)  -Mechanical trauma to the mouth (37%) | No information on this was provided. |
| 57 | Fayaz et al. (2024) | Afghanistan (Kabul) | University | Students (dental) and dentists (interns) | 453 | -60.7% were females  -42.6% were fifth-year dental students | -Tobacco chewing and smoking (74.6%) | No information on this was provided. |
| 58 | Kamal et al. (2024) | Afghanistan (Kabul) | Clinic | Patients | 435 | -All were aged between 15 and 76 years  -79.1% were males | -Family history (14.7%)  -Poor diet (22.5%)  -Poor oral hygiene (92.2%)  -Tobacco use (99.2%) | No information on this was provided. |
| 59 | Kanmodi et al. (2024b) | Nigeria (location not specified) | Clinic | Dentists | 75 | -Mean age was 45.61 years  -80% were males  -Mean years of clinical practice was 17.08 years | -Stem cell transplantation (1.3%)  -Eating of betel or areca nut (2.7%)  -Oral sex (6.7%)  -Family history of oral cancer (6.7%)  -Human papillomavirus infection (6.7%)  -Presence of premalignant lesion (14.7%)  -Poor diet (24.0%)  -Poor oral hygiene (33.3%)  -Low socioeconomic status (38.7%)  -Alcohol use (50.7%)  -Tobacco use (70.7%) | No information on this was provided. |
| 60 | Lee & Mun (2024) | Korea (location not specified) | Online | Dental hygienists | 180 | -Mean age was 29.9 years  -53.3% had associate’s degrees | -Exposure to the sun (42.5%)  -Old age (43.3%)  -Low consumption of vegetables and fruits (48.4%)  -Alcohol use (49.4%)  -Human papillomavirus infection (80.6%)  -Tobacco use (98.0%)  -Family history of oral cancer (98.4%) | No information on this was provided. |
| 61 | Mahdi (2024) | Iraq (Baghdad) | University | Students (medical) | 357 | -58.8% were aged 20 years or above  -60.8% were females | -Poor nutrition (0.3%)  -Eating of spicy food (1.1%)  -Alcohol use (5.6%)  -Genetic factors (16.2%)  -Viruses (27.5%)  -Smoking (49.3%) | No information on this was provided. |
| 62 | Shubayr et al. (2024) | Saudi Arabia (location not specified) | Online | Students (dental) and dentists | 157 | -Mean age was 26.3 years  -51.0% were males  -61.8% were dentist-clinicians | -Hereditary factor (59.9%)  -Alcohol and tobacco (69.4%)  -Older age (77.1%) | No information on this was provided. |

**Table S4. Summary of current primary studies reporting the prevalence and determinants of awareness and knowledge of the clinical features of oral cancer among diverse population groups**

| **No.** | **Author(s) (Year)** | **Country** | **Setting** | **Study Population** | **Sample Size** | **Sample Characteristics** | **Prevalence of Awareness/Knowledge of the Clinical Features of Oral Cancer** | **Determinants of Awareness/Knowledge of the Clinical Features of Oral Cancer** |
| --- | --- | --- | --- | --- | --- | --- | --- | --- |
| 1 | Alqahtani et al. (2020) | Saudi Arabia (Mecca) | Clinic | Patients | 416 | -71.4% were females  -57.7% were aged between 18 and 30 years  -44.5% had educational level at high school level or lower | -White or red oral patch (52.9%)  -Difficulty in swallowing or chewing (55.9%)  -Nonhealing oral sore (75.5%)  -Abnormal oral lump (77.5%) | No information on this was provided. |
| 2 | Bhat et al. (2020) | India (Bengaluru) | University | Students (non-health sciences) | 800 | -52% were males  -37.8% were in their third year of study | -Burning oral sensation (42.5-49%)  -Oral numbness (25-38%)  -Difficulty in chewing or swallowing (31.5-19%)  -Abnormal oral swelling (42.5-51.5%)  -Non-healing oral sore that bleeds easily (21.5-29%)  -White or red gingival patch (43-50.5%)  -Neck lump or thickening (44-48%) | No information on this was provided. |
| 3 | Gunjal et al. (2020) | Malaysia (Petaling Jaya) | University | Students (medical and dental) | 557 | -63.0% were medical students  -37.0% were dental students  -54.7% were aged 22 to 24 years  -63.4% were females | -Oral numbness (58.9%)  -Appearance as white lesion (70.6%)  -Appearance as mixture of red and white lesions (76.8%)  -Appearance as red lesion (78.6%)  -Oral swelling (80.3%) | No information on this was provided. |
| 4 | Nocini et al. (2020) | Italy (location not specified) | Online | Community dwellers and patients | 505 | -71.49% were females  -All were aged between 18 and 76 years  -44.75% had high school diploma | -Xerostomia (15%)  -Red lesions (20%)  -Burning oral sensation (28%)  -White lesions (37%) | No information on this was provided. |
| 5 | Oswal et al. (2020) | India (Assam, Nagaland, Meghalaya) | Community | Community dwellers | 1400 | -71.1% were aged 44 years or below  -49% were females  -62% had secondary school-level or above education | -Reduced quantity of saliva in the mouth (6%)  -Loss of taste sensation (12%)  -Toothache (19%)  -Difficulty in opening the mouth (30%)  -Dysphagia (37%)  -Neck swelling (40%)  -Multiple or nonhealing oral ulcers (51%) | No information on this was provided. |
| 6 | Pokhrel & Khadka (2020) | Nepal (Kathmandu) | University | Students (Dental) | 101 | -20.8% were males  -Mean age of all were 22.28 years  -48.5% were either in their third or fourth year | -Indurated oral ulcer (23.8%)  -Fixation of oral tissue (28.7%)  -Nonhealing chronic oral ulcer (47.5%)  -Red and white oral lesions (63.8%) | No information on this was provided. |
| 7 | Poudel et al. (2020) | Nepal (Kathmandu) | University | Students (dental) and dentists | 508 | -24.4% were in their third year of study  -3.1% were dentists | -Oral numbness (0.19%)  -Necrosis (0.19%)  -Chronic oral infection (0.39%)  -Pain (0.98%)  -Induration (1.18%)  -Lymphadenopathy (1.18%)  -Oral bleeding (1.96%)  -Oral mucosal hyperkeratosis (3.34%)  -Mobile tooth (3.54%)  -Red oral lesion (3.93%)  -White oral lesion (3.393%)  -Oral swelling (20.66%)  -Red and white oral lesion (26.18%)  -Colour change of oral mucosa (26.96%)  -nonhealing oral ulcer (39.37%) | No information on this was provided. |
| 8 | do Prado et al. 2020 | Brazil (Recife) | Clinic | Patients | 251 | -64.5% were aged 18 to 50 years  -64.9% were females  -16.7% had college-level education  -11.6% were smokers  -42.6% were alcohol users | -Loss of appetite (10.8%)  -Oral swelling and pain (17.6%)  -Painless oral swelling (22.3%)  -Change in voice (23.5%)  -Painful oral wound (28.7%)  -Bleeding of the gums (31.1%)  -Red oral patches (31.5%)  -White oral patches (33.1%)  -Painless nonhealing oral wound (83.3%) | No information on this was provided. |
| 9 | Shahabudin et al. (2020) | Malaysia (Kedah) | Community | Community dwellers | 96 | -54.2% were aged ≥54.2 years  -95.8% had secondary school education  -88.5% were females  -68.8% were married | -Nonhealing oral ulcer (51.0%)  -Gingival bleeding (53.1%)  -Oral red or white spot (54.2%) | No information on this was provided. |
| 10 | Singh et al. (2020) | India (Bareilly) | Community | Community dwellers | 800 | -38.9% were aged between 18 and 27 years  -62.3% were males  -43.9% had formal education up to the tenth class | -Painless swelling (5.1%)  -Change in voice (5.3%)  -Burning mouth sensation (6.1%)  -Red or white oral patch (8.9%)  -Dysphagia (11.9%)  -Inability to open the mouth (8.5%) | No information on this was provided. |
| 11 | Srivastava et al. (2020) | India (Kanpur) | University | Students (Dental) | 139 | -56.1% were interns  -56.8% were females | -Nonhealing oral ulcer (48.9%)  -Proliferative oral growth (51.1%) | No information on this was provided. |
| 12 | Yalcin & Gundogar (2020) | Turkey (Anatolia) | Clinic | Patients | 389 | -71.7% were aged ≤40 years  -53,7% were females  -41.4% had university-level education | -White oral patches (18.9%)  -Red oral patches (27.9%)  -Nonhealing oral ulcer (63%) | No information on this was provided. |
| 13 | Zachar et al. (2020) | Australia (Bathurst, Orange, Dubbo, Albury-Wodonga, and Wagga  Wagga) | Clinic | Patients | 444 | -70.2% were aged >40 years  -60.9% were females  -61.0% were employed  -14.7% were current smokers  -77.1% were current alcohol users | -Dental caries (29.3%)  -Infection of the tooth (34.3%)  -White oral patch (44.1%)  -Painless oral ulcer (44.1%)  -Red oral patch (44.6%)  -Odynophagia (53.8%)  -Oral bleeding (65.5%)  -Nonhealing oral ulcer (90.3%) | **Significant determinants:**  -Level of education (p=0.041)  **Non-significant determinants:**  -No information on this was provided. |
| 14 | Jafer et al. (2021) | Saudi Arabia (Jazan) | Clinic | Patients | 315 | -Mean age was 31 years  -58.8% were females  -45.7% had university level education | -Oral or peri-oral swellings (33.4%)  -Oral or peri-oral lumps (38.8%)  -Mobile teeth (52%)  -Oral or peri-oral sores (64.1%)  -Oral or peri-oral thick patches (76.3%) | -No information on this was provided. |
| 15 | Muthanandam et al. (2021) | India (Lawspet) | Community | Community dwellers | 329 | -53.2% were females  -39.2% were aged between 31 and 60 years  -91% were not formally educated | -Oral ulcer (31.94%)  -Red colour change (1.39%)  -Oral tissue growth (44.4%) | No information on this was provided. |
| 16 | Tavakoli et al. (2021) | United Kingdom (location not specified) | Clinic | Clinical staff (medical doctors, nurses, and healthcare assistants) | 155 | -11% were medical doctors  -61.3% were nurses  -27.7% were healthcare assistants | -Toothache (29%)  -Oral red or white patch (66%)  -Chronic oral ulcer (80%)  -Oral swelling or lump (86%) | No information on this was provided. |
| 17 | Al Hulaibi et al. (2022) | Saudi Arabia (Jazan) | Online | Community dwellers | 400 | -45.8% were singles  -64.9% were females  -80% were aged 32 years or more  -52% were married  -56.4% dwelled in the village | -Red spots in the mouth (32.8%)  -White spots in the mouth (36.1%)  -Chronic oral ulcer (53.1%)  -Oral lump (58.9%) | No information on this was provided. |
| 18 | Alzabibi et al. (2022) | Syria (Damascus) | University | Students (medical) | 301 | -All were aged between 18 and 30 years  -61.15% were males  -65.5% were in the clinical phase of their academic programme | -Oral bleeding (65.8%)  -Nonhealing oral ulcers (76.1%)  -Oral necrosis (73.1%)  -Oral lump (80.4%) | No information on this was provided. |
| 19 | de Lima Medeiros et al., 2022 | Brazil (locations not specified) | Online | Students (dental) and dentists | 623 | -Mean age of the participating dental students was 22.44 years while that of the dentists was 33.94 years  -81.2% of the dental students were females while 72% of the dentists were females | -Oedema (1-20%)  -Aphthous ulcers (2-10%)  -Gingival hyperplasia (4-16%)  -Oral tumour or nodule with a smooth surface (4-16%)  -Purple-coloured lesions (6-13%)  -Brown spots in the mouth (6-14%)  -Red and/or white oral spots (61%)  -Oral ulcer (65-70%)  -Oral tumour or nodule with ulcerated surface (72-73%) | No information on this was provided. |
| 20 | Gerber et al. (2022) | Poland (Łódź; Wrocław), Germany (Dresden) | Clinic | Patients | 454 | -Aged 18 to 95 years  -64.5% were females  -85.2% had secondary or tertiary level of education  -49.1% were single  -31.9% resided in the village | -Dysphagia (45.6%)  -White or red oral plaques (46.03%)  -Oral numbness (48.9%)  -Oral pain (50.2%)  -Oral lump (59.8%)  -Nonhealing oral wounds (60.8%) | No information on this was provided. |
| 21 | Jarab et al. (2022) | Jordan (location not specified) | Online | Community dwellers | 1307 | -Mean age was 26.10 years  -77.35% were females  -77.9% were not married  -90.1% had bachelor’s degree education or higher | -Changes in speech (52.02%)  -Neck lump (54.21%)  -Mobile teeth (59.67%)  -Pain in the jaw and/or ear (59.67%)  -Oral numbness (60.77%)  -Unexplained weight loss (65.46%)  -Chronic halitosis (65.57%)  -White or red oral patch (64.81%)  -Persistent sore throat (68.85%)  -Change in voice (69.51%)  -Oral bleeding or pain (74.97%)  -Nonhealing oral or lip sore (76.17%)  -Difficulty in jaw or tongue movement, chewing, or swallowing (80.87%)  -Lump in the throat, oral cavity, or lip (81.53%) | No information on this was provided. |
| 22 | Murariu et al. (2022) | Romania (Iasi) | University | Dentists and students (dental) | 197 | -Mean age was 25.01 years  -64.0% were females  -28.9% were resident dentists | -Abscess (6.6%)  -Oral ulcers (35.0%)  -Nodules with induration (55.8%) | No information on this was provided. |
| 23 | Saraswat et al. (2022) | Australia (eight states in Australia) | Online and phone | Community dwellers | 164 | -Mean age was 35.2 years  -52.8% were males  -47.5% were resident of New South Wales  -90% had university education | -Sore throat (29.9%)  -Oral pain or discomfort (41.5%)  -Painless oral ulcer (43.3%)  -Oral red discolouration or patch (46.3%)  -Oral white discolouration or patch (46.3%)  -Bleeding of the gums (48.8%)  -Nonhealing oral ulcer (57.9%) | No information on this was provided. |
| 24 | Taneja et al. (2022) | India (location not specified) | University | Dentists | 205 | -Mean age was 23.02 years  -57.2% were females | -Oral ulcer (42.9%)  -Painless, hard, mobile or fixed lymph node (43.7%) | No information on this was provided. |
| 25 | Yang et al. (2022) | Australia (Queensland) | Clinic | Patients | 213 | -51.3% were aged above 60 years  -54.4% were females  -53.8% were educated to tertiary level | -Mobile teeth (≈33%)  -White oral patch (≈35%)  -Reduced mouth opening (≈37%)  -Red oral patch (≈38%)  -Altered oral sensations (≈39%)  -Change in voice (≈40%)  -Loss of taste (≈42%)  -Odynophagia (≈45%)  -Difficulty in moving the tongue (≈49%)  -Bleeding of the gums (≈50%)  -Nonhealing oral ulcer (≈67%) | **Significant determinants:**  -Family history of oral cancer (p<0.05)  **Non-significant determinants:**  -No information on this was provided. |
| 26 | Zhou et al. (2022) | China (Beijing) | Community | Community dwellers | 3055 | -53.0% were aged 15 to 29 years  -54.2% were females  -53.7% were single  -81.5% had tertiary level education | -Red oral plaque (17.5%)  -White oral plaque (22.7%)  -Chronic nonhealing oral ulcers (40.0%) | **Significant determinants:**  -Age (p<0.05)  -Frequency of mouth self-examination practices (p<0.05)  -Place of residence (p<0.05)  -Level of income (p<0.05)  **Non-significant determinants:**  -No information on this was provided. |
| 27 | Anirudh et al. (2023) | India (specific location not mentioned) | Clinic | Patients | 158 | -61% were females  -39.2% were aged between 46 and 60 years  -46% had secondary school education | -Oral pain and bleeding (2.5%)  -Oral swelling (10.8%)  -Oral ulcers (20.3%)  -Elevated oral red and white patches (22.2%)  -Red or white oral patch (25.8%) | No information on this was provided. |
| 28 | Chatterjee et al. (2023) | India (Indore) | University | Students (homeopathy and ayurveda) | 310 | -Mean age was 23.84 years  -52.3% were females  -32.9% were in their third year of academic programme | -Red and/or white lesion (80.6%) | No information on this was provided. |
| 29 | Chugh et al. (2023) | India (Rajasthan) | Clinic | Patients | 446 | -83.6% were males  -63.1% were in the low socioeconomic status  -40.8% belonged to the age group of 21 to 30 years | -Suddenly mobile teeth (≈4%)  -Chronic oral swelling (≈5%)  -Nonhealing white or red oral patch (≈16%)  -Nonhealing oral ulcer (≈20%)  -Decrease in mouth opening (30%)  -Change in voice (≈55%) | No information on this was provided. |
| 30 | Shamala et al. (2023) | Yemen (Sana’a, Ibb, Aden, Taiz) | University | Students (dental) | 927 | -62% were females  -Mean age was 23.13 years  -33% were students of private universities | -Red lesion (64.8%)  -White lesion (68.8%)  -Speckled (red and white) lesion (76%)  -Oral bleeding (45.3%)  -Oral lump (81.6%)  -Nonhealing oral ulcer (84.1%) | No information on this was provided. |
| 31 | Uguru et al. (2023) | Nigeria (Enugu) | Military | Army personnel | 300 | -All were aged between 19 and 59 years  -Mean age was 37.5 years  -79.7% were males  -41.7% had tertiary level of education  -61.7% had 1 to 15 years’ experience in the army | -Sudden change in voice (14.7%)  -Red oral patch (15.7%)  -Sudden mobility of the tooth (16.7%)  -White oral patch (17.0%)  -Sudden difficulty in mouth opening (18.3%)  -Nonhealing extraction socket (19.3%)  -Nonhealing lip sore (22.0%)  -Nonhealing oral ulcer (25.0%)  -Oral or jaw swelling (25.7%) | No information on this was provided. |
| 32 | Dallak et al. (2024) | Saudi Arabia (Jazan) | Community | Community dwellers | 1380 | -52.9% were females  -63% were aged between 18 and 30 years  -45.7% were married  -50.6% lived in rural areas | The information below was concerning those participants (n=1022) who were aware of oral cancer:  -Tooth loss (30.5%)  -Oral red spots (32.8%)  -Difficulty in chewing or swallowing (38.9%)  -Gingival bleeding (41.4%)  -Nonhealing oral ulcers (45.0%)  -Oral pain (47.2%)  -Oral white spots (51.2%)  -Tumour or mass (62.1%)  -Oral ulcers (76.9%) | No information on this was provided. |
| 33 | Kamal et al. (2024) | Afghanistan (Kabul) | Clinic | Patients | 435 | -All were aged between 15 and 76 years  -79.1% were males | -Change in voice quality (14.5%)  -Oral sore (28.7%)  -Red or white oral patch (52%)  -Difficulty in chewing or swallowing (92%)  -Abnormal mass (98%) | No information on this was provided. |

**Table S5. Summary of the included primary studies which investigated the prevalence and determinants of knowledge of the preventability and preventative strategies of oral cancer among their study participants**

| **No.** | **Author(s) (Year)** | **Country** | **Setting** | **Study Population** | **Sample Size** | **Sample Characteristics** | **Prevalence of Awareness/Knowledge on Oral Cancer Preventability and Preventative Strategies** | **Determinants of Awareness/Knowledge on Oral Cancer Preventability and Preventative Strategies** |
| --- | --- | --- | --- | --- | --- | --- | --- | --- |
| 1 | Gunjal et al. (2020) | Malaysia (Petaling Jaya) | University | Students (medical and dental) | 557 | -63.0% were medical students  -37.0% were dental students  -54.7% were aged 22 to 24 years  -63.4% were females | -74.1% and 92.7% of the medical students and dental students, respectively, were aware that oral cancer is preventable  -No information on participant’s awareness/knowledge of oral cancer preventative strategies was provided. | No information on this was provided. |
| 2 | Nocini et al. (2020) | Italy (location not specified) | Online | Community dwellers and patients | 505 | -71.49% were females  -All were aged between 18 and 76 years  -44.75% had high school diploma | -No information on participant’s awareness/knowledge on the preventability of oral cancer  -11.68% knew how to do oral self-examination to detect oral cancer | No information on this was provided. |
| 3 | Pokhrel & Khadka (2020) | Nepal (Kathmandu) | University | Students (Dental) | 101 | -20.8% were males  -Mean age of all were 22.28 years  -48.5% were either in their third or fourth year | -No information on participant’s awareness/knowledge on the preventability of oral cancer  -30.7% reported that they had adequate knowledge on oral cancer preventative strategies | No information on this was provided. |
| 4 | Shahabudin et al. (2020) | Malaysia (Kedah) | Community | Community dwellers | 96 | -54.2% were aged ≥54.2 years  -95.8% had secondary school education  -88.5% were females  -68.8% were married | --No information on participant’s awareness/knowledge on the preventability of oral cancer  -55.2% identified oral self-examination as an oral cancer preventative strategy | No information on this was provided. |
| 5 | Srivastava et al. (2020) | India (Kanpur) | University | Students (Dental) | 139 | -56.1% were interns  -56.8% were females | -No information on participant’s awareness/knowledge on the preventability of oral cancer  -27.3% reported that they had adequate knowledge on oral cancer preventative strategies | No information on this was provided. |
| 6 | Jafer et al. (2021) | Saudi Arabia (Jazan) | Clinic | Patients | 315 | -Mean age was 31 years  -58.8% were females  -45.7% had university level education | -No information on participant’s awareness/knowledge on the preventability of oral cancer  -40.1%, 76.8%, 80.2%, and 83.8% of the participants reported that engagement in sporting activities, stopping smoking, avoiding smoking, and eating of vegetables are preventative strategies against oral cancer | No information on this was provided. |
| 7 | Muthanandam et al. (2021) | India (Lawspet) | Community | Community dwellers | 329 | -53.2% were females  -39.2% were aged between 31 and 60 years  -91% were not formally educated | -16.67% were aware that oral cancer is preventable  --No information on participant’s awareness/knowledge on the preventative strategies of oral cancer | No information on this was provided. |
| 8 | Wong et al. (2021) | United States of America (Florida) | Telephone | Community dwellers | 2260 | -Mean age was 55.9 years  -55.8% were females  -70.6% were Caucasians | -No information on participant’s awareness/knowledge on the preventability of oral cancer  -46.8% were aware of oral cancer examination | No information on this was provided. |
| 9 | Adeoye et al. (2022) | China (Hong Kong) | Online | Community dwellers | 964 | -Aged between 18 and 86 years  -58.2% were females  -65.0% had tertiary level education  -6.4% were current tobacco users  -14.8% were former tobacco users  -15.7% were current alcohol drinkers  -7.0% were former alcohol drinkers | -No information on participant’s awareness/knowledge on the preventability of oral cancer  -61.4% were aware of oral cancer screening | No information on this was provided. |
| 10 | Al Hulaibi et al. (2022) | Saudi Arabia (Jazan) | Online | Community dwellers | 400 | -45.8% were singles  -64.9% were females  -80% were aged 32 years or more  -52% were married  -56.4% dwelled in the village | -69.3% were aware that oral cancer is preventable  -72.7% were aware of oral cancer screening | No information on this was provided. |
| 11 | Shadid et al. (2022) | Palestine (West Bank) | University | Dental interns and students (dental) | 392 | -94.1% were aged 30 years or below  -64.3% were females  -10.5% were interns | -No information on participant’s awareness/knowledge on the preventability of oral cancer  -68.1% reported to have adequate knowledge (training) on tobacco cessation as an oral cancer preventative strategy  -62.3% reported to have adequate knowledge (training) on alcohol cessation as an oral cancer preventative strategy | No information on this was provided. |
| 12 | Zhou et al. (2022) | China (Beijing) | Community | Community dwellers | 3055 | -53.0% were aged 15 to 29 years  -54.2% were females  -53.7% were single  -81.5% had tertiary level education | -38.2% were aware that oral cancer is preventable  -46.2% were aware that change of habits or lifestyle can reduce the risk of developing oral cancer | No information on this was provided. |
| 13 | Dixit et al. (2023) | India (location not specified) | University | Students (dental) and dentists (interns) | 300 | -94.1% were aged 30 years or below  -64.5% were females  -65.4% were fifth-year dental students | -65.1% had poor knowledge on oral cancer prevention  -No specific information was provided on participant’s awareness/knowledge on oral cancer preventability or preventative strategies | No information on this was provided. |
| 14 | Shadid & Habash (2023) | Palestine (locations not specified) | Clinical | Dentists | 254 | -65.7% were aged above 30 years  -43.7% were females  -42.5% had 6 to 15 years of experience  -80.7% worked in a private setting  -79.9% were general dentists | -70.1% had poor knowledge on oral cancer prevention, including oral cancer screening  -No specific information was provided on participant’s awareness/knowledge on oral cancer preventability or preventative strategies | No information on this was provided. |
| 15 | Dallak et al. (2024) | Saudi Arabia (Jazan) | Community | Community dwellers | 1380 | -52.9% were females  -63% were aged between 18 and 30 years  -45.7% were married  -50.6% lived in rural areas | The information below was concerning those participants (n=1022) who were aware of oral cancer:  -44.7% were aware that oral cancer is preventable  -No specific information was provided on participant’s awareness/knowledge on oral cancer preventative strategies | No information on this was provided. |
| 16 | Das et al. (2024) | India (Odisha) | University | Students (nursing, medical, and dental) | 1000 | -23%, 35.4%, and 41.6% were nursing, medical, and dental students respectively | -No information on participant’s awareness/knowledge on the preventability of oral cancer  -61.5% were aware that vaccination against human papillomavirus can prevent oral cancer, and no information was provided on participant’s awareness/knowledge on other oral cancer preventative strategies | No information on this was provided. |
| 17 | Shubayr et al. (2024) | Saudi Arabia (location not specified) | Online | Students (dental) and dentists | 157 | -Mean age was 26.3 years  -51.0% were males  -61.8% were dentist-clinicians | -24.2% were aware that oral cancer is preventable  -No specific information was provided on participant’s awareness/knowledge on oral cancer preventative strategies | No information on this was provided. |

**Table S6. Summary of current primary studies reporting the prevalence and determinants of awareness and knowledge on oral cancer diagnostic approaches**

| **No.** | **Author(s) (Year)** | **Country** | **Setting** | **Study Population** | **Sample Size** | **Sample Characteristics** | **Prevalence of Awareness/Knowledge on Oral Cancer Diagnostic Approaches** | **Determinants of Awareness/Knowledge on Oral Cancer Diagnostic Approaches** |
| --- | --- | --- | --- | --- | --- | --- | --- | --- |
| 1 | Gunjal et al. (2020) | Malaysia (Petaling Jaya) | University | Students (medical and dental) | 557 | -63.0% were medical students  -37.0% were dental students  -54.7% were aged 22 to 24 years  -63.4% were females | -31.9% and 72.8% of the medical students and dental students, respectively, were reported to have knowledge on oral cancer diagnostic approaches  -No specific information was provided on the types of oral cancer diagnostic approaches known by the participants | No information on this was provided. |
| 2 | Poudel et al. (2020) | Nepal (Kathmandu) | University | Students (dental) and dentists | 508 | -24.4% were in their third year of study  -3.1% were dentists | -8.9% and 33.3% of the participating third-year dental students and dentists, respectively, had knowledge on oral cancer diagnostic approaches  -No specific information was provided on the types of oral cancer diagnostic approaches known by the participants |  |
| 3 | Srivastava et al. (2020) | India (Kanpur) | University | Students (Dental) | 139 | -56.1% were interns  -56.8% were females | -27.3% had sufficient knowledge on oral cancer diagnostic approaches  -No specific information was provided on the types of oral cancer diagnostic approaches known by the participants |  |
| 4 | Ojha et al. (2021) | Nepal (Kathmandu) | Clinic | Dentists | 234 | -68.5% were females  -Mean age was 27 years | -59.4% were reported to have knowledge on oral cancer diagnostic approaches  -The diagnostic approaches reported were biopsy, and the use of toluidine blue (7/234; 3.0%) | No information on this was provided. |
| 5 | Wimardhani et al. (2021) | Indonesia (Jakarta) | Clinic | Dentists | 402 | -79.6% were females  -37.8% were aged between 30 and 49 years  -22.9% were from the eastern region of Jakarta | -None (0%) had good knowledge on oral cancer diagnostic approaches | No information on this was provided. |
| 6 | Jarab et al. (2022) | Jordan (location not specified) | Online | Community dwellers | 1307 | -Mean age was 26.10 years  -77.35% were females  -77.9% were not married  -90.1% had bachelor’s degree education or higher | -Barium swallow (27.54%)  -Panoramic radiographs (35.19%)  -Endoscopy (59.67%)  -Blood tests (66.56%)  -Computed tomography scan (60.55%)  -Magnetic resonance imaging (72.68%)  -Oral brush biopsy (75.08%) | No information on this was provided. |
| 7 | Tarakji (2022) | Saudi Arabia (multiple universities) | University | Students (dental) | 189 | -65% were males  -70% were aged 17 to 23 years  -72% were attending private universities | ->79% were reported to knew biopsy as an oral cancer diagnostic approach  -No information on other oral cancer diagnostic approaches was provided | No information on this was provided. |
| 8 | Chan et al. (2023) | Malaysia (13 dental schools in Malaysia) | Online | Students (dental) | 595 | -56% were females  -77.1% wee aged between 22 and 24 years | -74.1% were reported to have knowledge on oral cancer diagnostic approaches  -No specific information was provided on the types of oral cancer diagnostic approaches known by the participants | No information on this was provided. |

**Table S7. Summary of current primary studies reporting the prevalence and determinants of awareness and knowledge on the treatability and treatment options of oral cancer**

| **No.** | **Author(s) (Year)** | **Country** | **Setting** | **Study Population** | **Sample Size** | **Sample Characteristics** | **Prevalence of Awareness/Knowledge on Oral Cancer Treatability and its Treatment Options** | **Determinants of Awareness/Knowledge on Oral Cancer Treatability and its Treatment Options** |
| --- | --- | --- | --- | --- | --- | --- | --- | --- |
| 1 | Alqahtani et al. (2020) | Saudi Arabia (Mecca) | Clinic | Patients | 416 | -71.4% were females  -57.7% were aged between 18 and 30 years  -44.5% had educational level at high school level or lower | -78.4% were aware that oral cancer is treatable  -No information was provided concerning the participants’ awareness or knowledge of oral cancer treatment options. | No information on this was provided. |
| 2 | Yalcin & Gundogar (2020) | Turkey (Anatolia) | Clinic | Patients | 389 | -71.7% were aged ≤40 years  -53,7% were females  -41.4% had university-level education | -69.3% were aware that oral cancer is treatable  -No information was provided concerning the participants’ awareness or knowledge of oral cancer treatment options. | No information on this was provided. |
| 3 | Tavakoli et al. (2021) | United Kingdom (location not specified) | Clinic | Clinical staff (medical doctors, nurses, and healthcare assistants) | 155 | -11% were medical doctors  -61.3% were nurses  -27.7% were healthcare assistants | -19.3% reported that the treatability of oral cancer was ≤25% while others reported it to be >25%  -100%, 95%, and 94% of the participating medical doctors, healthcare assistants, and nurses, respectively, identified oral and maxillofacial surgeons as specialists that manage oral cancer  -98%, 91%, 88% of healthcare assistants, nurses, and doctors, respectively, identified oncologists as specialists that manage oral cancer | No information on this was provided. |
| 4 | Muthanandam et al. (2021) | India (Lawspet) | Community | Community dwellers | 329 | -53.2% were females  -39.2% were aged between 31 and 60 years  -91% were not formally educated | -30.56% were aware that oral cancer is treatable, if diagnosed early  -37.5%, 16.67%, and 5.56% were aware of the use of medicines, surgery, or both (i.e. the use of medicines and surgery) as management options for oral cancer | No information on this was provided. |
| 5 | Al Hulaibi et al. (2022) | Saudi Arabia (Jazan) | Online | Community dwellers | 400 | -45.8% were singles  -64.9% were females  -80% were aged 32 years or more  -52% were married  -56.4% dwelled in the village | -86% were aware that oral cancer is treatable, if diagnosed early  -59.8%, 56.3%, and 55.0% were aware that chemotherapy, radiotherapy, and surgery are the treatment options of oral cancer | No information on this was provided. |
| 6 | Alzabibi et al. (2022) | Syria (Damascus) | University | Students (medical) | 301 | -All were aged between 18 and 30 years  -61.15% were males  -65.5% were in the clinical phase of their academic programme | -75% were aware that oral cancer is treatable, if diagnosed early  -67.1%, 21.3%, 9.6%, and 2% were aware that oncologists, otorhinolaryngologists, oral and maxillofacial surgeons, and dentists, respectively can treat oral cancer | No information on this was provided. |
| 7 | Zhou et al. (2022) | China (Beijing) | Community | Community dwellers | 3055 | -53.0% were aged 15 to 29 years  -54.2% were females  -53.7% were single  -81.5% had tertiary level education | -62.3% were unaware of the treatment options available for oral cancer ->70% were unaware of the therapeutic effect of oral cancer treatment  ->70% were unaware of the costs of oral cancer treatment options | No information on this was provided. |
| 8 | Chan et al. (2023) | Malaysia (13 dental schools in Malaysia) | Online | Students (dental) | 595 | -56% were females  -77.1% wee aged between 22 and 24 years | -90.6% were aware that oral cancer is treatable  -No information was provided concerning the participants’ awareness or knowledge of oral cancer treatment options. | No information on this was provided. |
| 9 | Chugh et al. (2023) | India (Rajasthan) | Clinic | Patients | 446 | -83.6% were males  -63.1% were in the low socioeconomic status  -40.8% belonged to the age group of 21 to 30 years | -No information was provided concerning the participants’ awareness or knowledge on the treatability of oral cancer  -70% were aware that oral cancer can be treated using medicines, surgery, and radiotherapy | No information on this was provided. |
| 10 | Deshpande et al. (2023) | India (Sawangi) | Clinic | Healthy relatives of cancer patients | 400 | -52.5% were aged below 35 years  -50.5% were females  -62.3% were married  -47.5% were university graduates | -66.0% were aware that oral cancer is treatable  -42.7% were aware of the treatment options of oral cancer  -42.8% were aware that oral cancer can recur after treatment  -37.25% were aware of oral cancer chemotherapy | No information on this was provided. |
| 11 | Uguru et al. (2023) | Nigeria (Enugu) | Military | Army personnel | 300 | -All were aged between 19 and 59 years  -Mean age was 37.5 years  -79.7% were males  -41.7% had tertiary level of education  -61.7% had 1 to 15 years’ experience in the army | -72.7% were aware that oral cancer is treatable  -No information was provided concerning the participants’ awareness or knowledge of oral cancer treatment options. | No information on this was provided. |
| 12 | Dallak et al. (2024) | Saudi Arabia (Jazan) | Community | Community dwellers | 1380 | -52.9% were females  -63% were aged between 18 and 30 years  -45.7% were married  -50.6% lived in rural areas | The information below was concerning those participants (n=1022) who were aware of oral cancer:  -58% were aware that oral cancer is treatable  -No specific information was provided on participant’s awareness/knowledge on oral cancer treatment options | No information on this was provided. |
| 13 | Fayaz et al. (2024) | Afghanistan (Kabul) | University | Students (dental) and dentists (interns) | 453 | -60.7% were females  -42.6% were fifth-year dental students | -60.9% were aware that oral cancer is treatable  -No information was provided concerning the participants’ awareness or knowledge of oral cancer treatment options. | No information on this was provided. |
